# Elevated p16Ink4a Expression Enhances Tau Phosphorylation in Neurons Differentiated From Human‐Induced Pluripotent Stem Cells

**DOI:** 10.1111/acel.14472

**Published:** 2025-01-05

**Authors:** Kristopher Holloway, Kashfia Neherin, Yingduo Song, Kazuhito Sato, Andrew Houston, Feng Chen, Li Ding, Hong Zhang

**Affiliations:** ^1^ Department of Pediatrics, 3 NeuroNexus Institute University of Massachusetts Chan Medical School Worcester Massachusetts USA; ^2^ Department of Medicine, McDonnell Genome Institute Washington University School of Medicine St. Louis Missouri USA

**Keywords:** aging, Alzheimer's disease, cellular senescence, disease modeling, p16Ink4a, tau

## Abstract

Increased expression of the cyclin‐dependent kinase inhibitor p16Ink4a (p16) is detected in neurons of human Alzheimer's disease (AD) brains and during normal aging. Importantly, selective eliminating p16‐expressing cells in AD mouse models attenuates tau pathologies and improves cognition. But whether and how p16 contributes to AD pathogenesis remains unclear. To address this question, we tested whether induction of p16 expression in neurons exacerbates AD pathologies. We created a doxycycline‐inducible system to trigger p16 up‐regulation in human‐induced pluripotent stem cells (iPSCs) and neurons differentiated from iPSCs. We demonstrated that up‐regulated p16 expression in iPSCs reduces cell proliferation, down‐regulates cell cycle genes, and up‐regulates genes involved in focal adhesion, interferon α response and PI3K‐Akt signaling. Our approach enables temporal control of p16 induction upon differentiation from iPSCs to neurons. In differentiated cortical neurons, we found that up‐regulation of p16 increases tau phosphorylation at Ser202/Thr205 and Thr231 in a cell‐autonomous manner, while amyloid beta secretion is not affected. These data suggest a critical role of p16 in regulating tau phosphorylation in neurons, and thereby contributing to pathological progression of AD. As pathological tau tangles have been shown to induce p16 expression, our studies suggest a positive feedback loop between p16 and tau to exacerbate tau pathologies.

## Graphical abstract

We created an inducible system to control the expression of p16, and found that tau phosphorylation was enhanced upon p16 up‐regulation in neurons differentiated from human iPSCs. As p16 expression is increased during aging, our findings suggest a possible role of age‐associated p16 up‐regulation in Alzheimer's disease. As pathological tau tangles have been shown to induce p16 expression, our studies further suggest a positive feedback loop between p16 and tau to exacerbate tau pathology.

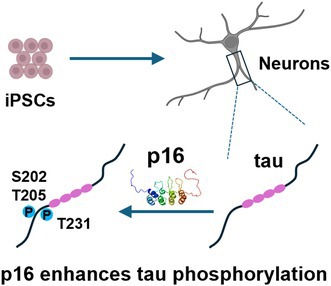

**Abbreviations**
AD: Alzheimer's disease
Aβ: amyloid‐beta
CDK: cyclin‐dependent kinase
DDR: DNA damage response
DEGs: differentially expressed genes
Dox: doxycycline
ECM: extracellular matrix
GSEA: gene set enrichment analysis
IF: immunofluorescence
iPSCs: induced pluripotent stem cells
MSigDB: Molecular Signature Database
NFT: neurofibrillary tangle
NPCs: neural progenitor cells
p16: p16Ink4a (CDKN2A)
rtTA: reverse tetracycline‐controlled transactivator
SASP: senescence‐associated secretory phenotype
SA‐β‐gal: senescence‐associated β‐galactosidase
STR: short tandem repeat

## 1 Introduction

Cellular senescence plays important roles in many physiological and pathological processes, including cancer, aging, and age‐associated diseases (Campisi 2013; van Deursen 2014). Recent studies have found that senescence plays an important role in the pathogenesis of Alzheimer's disease (AD) (Bhat et al. 2012; Bussian et al. 2018; Herdy et al. 2022; Hu et al. 2021; Musi et al. 2018; Streit et al. 2009; Zhang et al. 2019). Senescent cells including astrocytes, microglia, neurons, and oligodendrocyte precursor cells are found in the brains of human AD patients and AD mouse models (Arendt et al. 1996; Bhat et al. 2012; Bussian et al. 2018; Dehkordi et al. 2021; Gaikwad et al. 2021; Herdy et al. 2022; Hu et al. 2021; McShea et al. 1997; Musi et al. 2018; Streit et al. 2009; Zhang et al. 2019). As a senescence regulator, cyclin‐dependent kinase (CDK) inhibitor p16Ink4a (p16) is frequently up‐regulated in senescent cells (Campisi 2005; Collado, Blasco, and Serrano 2007), and up‐regulation of p16 in human fibroblasts has been found to activate cellular senescence (Coppe et al. 2011; Freund et al. 2012; McConnell et al. 1998). Further, increased p16 expression is reported in various types of cells (astrocytes, microglia, oligodendrocyte precursor cells, and neurons) in AD mouse models and human post‐mortem AD brains (Arendt et al. 1996; Bhat et al. 2012; Bussian et al. 2018; Dehkordi et al. 2021; Gaikwad et al. 2021; Herdy et al. 2022; McShea et al. 1997; Musi et al. 2018; Wei et al. 2016; Zhang et al. 2019). More importantly, selective clearing of p16‐expressing cells with a genetic approach or eliminating senescent cells using senolytic compounds in AD mouse models have been shown to ameliorate various aspects of AD pathologies including tau phosphorylation, neurofibrillary tangles (NFTs) formation, Aβ load, and cognitive decline (Bussian et al. 2018; Hu et al. 2021; Musi et al. 2018; Zhang et al. 2019). These findings suggest a critical role of senescence and p16 in AD pathogenesis, but the mechanisms underlying their roles in AD etiopathology remains unclear.

Although cellular senescence is commonly associated with cell cycle arrest accompanied by increased expression of p16 and/or another CDK inhibitor p21 in proliferating cells, recent evidence indicates that cellular senescence can occur in post‐mitotic cells (Farr et al. 2016; Minamino et al. 2009; Sapieha and Mallette 2018). Characteristic features of cellular senescence including p16 expression have been reported in non‐dividing neurons (Acklin et al. 2020; Dehkordi et al. 2021; Herdy et al. 2022; Jurk et al. 2012; Kang et al. 2015; Moreno‐Blas et al. 2019; Musi et al. 2018; Ohashi et al. 2018; Paramos‐de‐Carvalho et al. 2021; Raffaele et al. 2020; Riessland et al. 2019). Furthermore, neurons expressing senescence markers such as p16 are found to be more common in the prefrontal cortex of AD patients than cognitively normal controls, suggesting that AD neurons are prone to senescence (Herdy et al. 2022). Consistent with this notion, senescent neurons constitute the most senescent cells in AD brains (Dehkordi et al. 2021). Increased p16 expression is detected in neurons around amyloid beta (Aβ) plaques or tau tangle‐bearing neurons in the brains of human AD patients and AD mouse models (Arendt et al. 1996; Herdy et al. 2022; McShea et al. 1997; Musi et al. 2018; Wei et al. 2016). It has been shown that p16 expression can be induced by Aβ peptides and tau tangles in neurons (Musi et al. 2018; Wei et al. 2016). As a CDK inhibitor, p16 blocks the CDK4/CDK6‐Cyclin D complex from phosphorylation of the retinoblastoma (Rb) family of proteins. In its unphosphorylated state, Rb is active and binds to E2F transcription factors, preventing them from activating genes necessary for S phase entry and cell cycle progression (Sherr and Roberts 1999). It is not clear whether increased p16 expression in senescent neurons is merely a consequence of senescence or plays a contributing role in senescence. It is also not known whether the role of p16 in neuronal senescence is related to its function as a CDK inhibitor.

In this study, we directly investigated the impact of up‐regulation of p16 in human‐induced pluripotent stem cells (iPSCs) and iPSC‐derived cortical neurons on senescence and the development of AD pathologies. Human iPSC‐derived neurons have provided insight into AD phenotypes including increased Aβ secretion and tau phosphorylation (Israel et al. 2012; Penney, Ralvenius, and Tsai 2020; Sullivan and Young‐Pearse 2017; Yagi et al. 2011). We generated an inducible system to allow isogenic comparisons in cells with or without p16 expression. Using our system, we demonstrated that up‐regulation of p16 expression led to suppression of cell proliferation in iPSCs, accompanied by down‐regulation of cell cycle genes and up‐regulation of genes involved in focal adhesion, interferon α response and PI3K‐Akt pathways. Interestingly, we found that up‐regulation of p16 expression increases tau phosphorylation at Ser202/Thr205 and Thr231 in iPSC‐derived cortical neurons, indicating that p16 expression exacerbates neurodegenerative processes associated with AD in human neurons.

## 2 Materials and Methods

### 2.1 Pluripotent Stem Cell Lines and Cell Culture

A human iPSC line with the PSEN1‐A246E mutation (AG25367, fAD) was reprogrammed from skin fibroblasts of a 45‐year‐old familial AD patient (Lavekar et al. 2023). A human iPSC line with the APOE4/E4 variant (AG27609, sAD) was reprogrammed from skin fibroblasts of an 87‐year‐old sporadic AD patient (Meyer et al. 2019). Both lines were obtained from the Coriell Institute for Medical Research. A human iPSC line (35L11, Ctrl) was reprogrammed from skin fibroblasts of a healthy individual with no history of dementia (Freibaum et al. 2015) and generously provided by Dr. Fen‐Biao Gao of University of Massachusetts Chan Medical School. All iPSC lines were cultured in plates coated with recombinant human Vitronectin protein (Gibco) and fed daily with TeSR‐E8 medium (STEMCELL Technologies) at 37°C with 5% CO_2_. When iPSC cultures reached 70%–80% confluency, iPSC colonies were treated with 0.5 mM EDTA (Corning) at room temperature and passaged at 1:6 ratio. For single cell passaging, iPSC colonies were dissociated using TrypLE Express (Gibco) at 37°C for 3 min. To promote cell survival upon single cell passaging, 10 μM ROCK inhibitor Y‐27632 (R&D Systems) was added in the culture media for the first 24 h. All iPSC lines were regularly tested for mycoplasma using the Mycoplasma PCR Detection Kit (Applied Biological Materials).

### 2.2 Constructing of the Inducible p16 Expression Vector pAAVS1‐p16

The p16 cDNA with the codon‐optimized open reading frame was amplified from the pLX401‐INK4A vector (Addgene) (Howard et al. 2019) using Phusion High‐Fidelity DNA polymerase (NEB), and cloned into vector pAAVS1‐Ndi‐CRISPRi (Gen2) (Addgene) under the control of the TRE3G promoter (Gonzalez et al. 2014; Mandegar et al. 2016). Primer sequences are listed in Table S1.

### 2.3 Nucleofection of iPSCs and Junction PCR

Two million live singularized iPSCs were mixed with 5 μg pAAVS1‐p16 plasmid and 2 μg each of AAVS1‐TALEN‐L and AAVS1‐TALEN‐R plasmids (Addgene) (Gonzalez et al. 2014). Nucleofection was performed using the Nucleofector kit 2 and the Amaxa Nucleofector 2b device (Lonza; Program A‐23). Starting 48 h post‐nucleofection, cells were selected with G418 sulfate (Life Technologies). Stable colonies were individually picked under microscope and expanded with continuous G418 selection. Genomic DNA was extracted from isolated iPSC clones using the DNeasy blood and tissue kit (Qiagen) following the manufacturer's protocol. A quantity of 200 ng of genomic DNA was used to amplify 5′ or 3′ junctions around the AAVS1 integration sites and the wild‐type AAVS locus using the GoTaq polymerase master mix (Promega). Primer sequences are listed in Table S2.

### 2.4 Karyotyping and Short Tandem Repeat (STR) Analysis of iPSCs

Cytogenic analysis of samples from isolated iPSC clones was conducted by WiCell Research Institute. G‐band karyotyping was analyzed for at least 20 metaphases and STR analysis of 15 loci was carried out to verify the identity and purity of newly generated iPSC clones.

### 2.5 Neuron Differentiation of iPSCs

Neuron differentiation was performed as previously described with some modifications (Autar et al. 2022; Cao et al. 2017; Chambers et al. 2009; Czerminski and Lawrence 2020; Ruden et al. 2021). Briefly, iPSCs were dissociated into single cells using TrypLE Express and seeded at a density of 50,000 cells/well in a Vitronectin‐coated 24‐well plate in TeSR‐E8 media supplemented with 10 μM ROCK inhibitor Y‐27632. The next day, the media was changed with fresh TeSR‐E8 media. Two days post seeding, cells were fed daily with neural differentiation media (NDM) consisting of 50% Knockout DMEM/F12, 50% Neurobasal, 0.5X Glutamax, 1X N‐2 Supplement (all from Life Technologies) and supplemented with 2 μM DMH1 and 2 μM SB431542 (both from Tocris Bioscience). After 14 days, cells were broken into clumps after 0.5 mM EDTA treatment and cultured in suspension in non‐treated T75 flasks (USA Scientific) in NDM for 7 days. On day 21 of neural differentiation, neurospheres were dissociated into single cells using TrypLE Express and plated on coverslips (Electron Microscopy Sciences) coated with 15 μg/mL poly‐L‐ornithine and laminin (both from Sigma) in 24‐well plates with Knockout DMEM/F12 with 15 mM HEPES at a density of 30,000–50,000 cells/well, and fed every 2 days with neuron media consisting of Neurobasal, 1X N‐2, 0.5X B‐27 without vitamin A, 1X penicillin/streptomycin, 1X Glutamax (all from Life Technologies), 0.3% Glucose (Sigma), 20 ng/mL GDNF (Peprotech), 20 ng/mL BDNF (Peprotech), 35.224 μg/mL ascorbic acid, 1 μM cyclic AMP, and 1 μg/mL laminin (Sigma). Doxycycline hyclate (Sigma) was added to the culture media every other day starting on neural differentiation day 22 after breaking up neurospheres.

### 2.6 Immunofluorescence (IF) Staining and Imaging

Cultured cells grown on coverslips were fixed in 4% paraformaldehyde in PBS for 10 min at room temperature. Fixed cells were washed with PBS three times, permeabilized with 0.25% Triton X‐100 in PBS at room temperature for 10 min, and blocked in buffer containing 1% bovine serum albumin, 22.52 mg/mL glycine, and 0.1% Tween‐20 in PBS (PBST) (Sigma). Incubation of primary antibodies in PBST with 1% bovine serum albumin were performed at 4°C overnight. After washing with PBS three times, cells were incubated with fluorophore conjugated secondary antibodies at room temperature for 1 h. After washing with PBS three times, nuclei were counterstained with DAPI (BioLegend), and coverslips were mounted with Vectashield PLUS (Vector Laboratories). Detailed primary and secondary antibody information is listed in Table S3. Microscopy was performed using Zeiss Axio Observer 7 or Leica DMI6000B microscopes. Minimal correction for brightness and contrast were performed using ZEN software to acquire representative image signals. Cell counts, nuclei size measurements and integrated fluorescence intensities were measured using CellProfiler image analysis software (Stirling et al. 2021). For scoring and quantification, we examined at least three random fields in three independent differentiations of each cell line per condition.

### 2.7 Western Blot Analysis

Cells were lysed in RIPA buffer (150 mM NaCl, 5 mM EDTA, 50 mM Tris–HCl, 1% NP‐40, 0.5% sodium deoxycholate, 0.1% SDS) containing Complete protease inhibitor cocktails and phosSTOP phosphatase inhibitor (Roche). A total of 30 μg of lysate was loaded onto 4%–12% Criterion XT Bis‐Tris gel and transferred to nitrocellulose membrane (both from Bio‐Rad). Transfer of proteins was confirmed by Ponceau S (Sigma) staining. The membrane was blocked at room temperature for 1 h with 5% non‐fat dry milk in PBST. Primary antibody was diluted in 5% milk in PBST and incubated at 4°C overnight, followed by three washes with PBST. Then the membrane was incubated with HRP‐conjugated secondary antibody diluted in 5% milk in PBST at room temperature for 1 h. Blots were imaged using Clarity Western ECL reagents with the ChemiDoc imaging system (both from Bio‐Rad). Quantification of protein bands were performed using ImageJ and Bio‐Rad Image Lab software. Detailed primary and secondary antibody information is provided in Table S3.

### 2.8 RNA Sequencing Analysis

Total RNA was extracted with TRI Reagent (Sigma) and treated with DNase I using RNeasy MinElute Cleanup Kit (Qiagen). RNA integrity was determined using Agilent 4200 TapeStation. One microgram of total RNA was used to perform library preparation. Ribosomal RNA was removed using the RiboErase kit (Kapa Biosystems) and mRNA was then fragmented. First and second strand cDNA synthesis was performed using SuperScript III reverse transcriptase (Life Technologies) with random hexamers per manufacturer's instructions. Double stranded cDNA was ligated to Illumina sequencing adapters and then amplified for 15 cycles using primers incorporating unique dual index tags. Fragments were sequenced on Illumina NovaSeq‐6000 using paired end reads extending 150 bases targeting 30 M clusters. Gene‐level read counts, Fragments Per Kilobase of transcript per Million mapped reads (FPKM), FPKM Upper Quartile (FPKM‐UQ), and Transcripts Per Million (TPM) values were acquired using the HTAN Bulk RNA Expression pipeline developed by the Ding lab (https://github.com/ding‐lab). This pipeline, implemented with STAR v2.7.10b and SAMtools v1.15.1, generated aligned BAM files from raw FASTQ files (Dobin et al. 2013; Li et al. 2009). Read counts were obtained using featureCounts in Subread v2.0.3 and then converted to FPKM, FPKM‐UQ, and TPM using the formulas outlined in GDC's expression mRNA Pipeline documentation (https://docs.gdc.cancer.gov/Data/Bioinformatics_Pipelines/Expression_mRNA_Pipeline/; Liao, Smyth, and Shi 2014). For differential gene expression analysis, DESeq2 version 1.42.0 was used and a cutoff of absolute log_2_FoldChange > 0.585 (FC ≥ 1.5) and FDR (adjusted *p*‐value) < 0.05 was used to define differentially expressed genes (DEGs) (Love, Huber, and Anders 2014). Enrichment of KEGG pathways was performed using DAVID functional annotation tool (Fabregat et al. 2017; Huang da, Sherman, and Lempicki 2009a, 2009b). Gene Set Enrichment Analysis (GSEA) was performed using GSEA software v4.3.2 with 1000 permutations for gene sets and Signal2Noise metric for ranking genes (Subramanian et al. 2005). RNA‐seq data were deposited in the Gene Expression Omnibus (GSE252132).

### 2.9 5‐Ethynyl‐2′‐Deoxyuridine (EdU) Labeling and Cell Growth Curve Assay

Cells were treated with 3 μM EdU for 6 h at 37°C, followed by detection of EdU using Click‐iT EdU Alexa Fluor 594 cell proliferation kit (Invitrogen). After counterstaining with DAPI, cells were imaged by fluorescence microscopy. For growth curve assay, cell counting was performed daily for 4 days after plating using Countess II automated cell counter (Invitrogen).

### 2.10 Senescence‐Associated β‐Galactosidase (SA‐β‐Gal) Staining

Following fixation with 4% paraformaldehyde in PBS for 3 min, cells were stained using the SPiDER‐β‐Gal senescence cell detection kit (Dojindo laboratories) with incubation for 15 min at 37°C (Kim et al. 2019).

### 2.11 Statistical Analysis

All statistical analyses were performed, and graphs were generated using GraphPad Prism 10. Data were presented as mean ± standard deviation (SD). The Mann–Whitney *U* test was used for nonparametric pairwise sample comparison. For dose response, data were plotted with nonlinear regression. One‐way ANOVA for groups of three or more were followed by a Kruskal–Wallis multiple comparison test. *p* < 0.05 was considered statistically significant.

## 3 Results

### 3.1 Establishing Inducible p16 Expression in iPSCs

**FIGURE 1:**
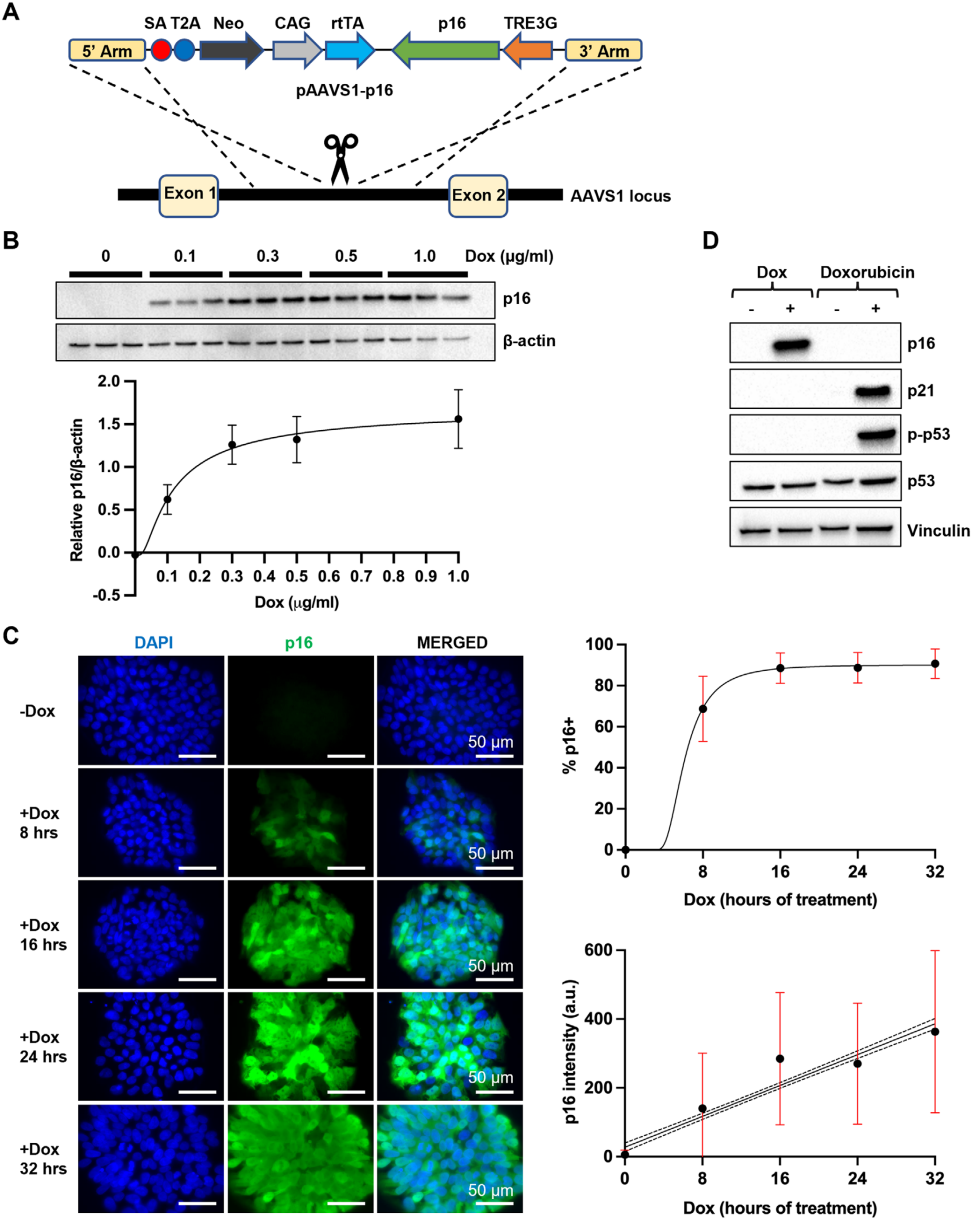
A doxycycline‐inducible system to up‐regulate p16 expression in human iPSCs. (A) Schematic overview of TALEN‐assisted targeting of p16 into the AAVS1 locus. (B) Dose response of p16 expression to 2‐day Dox treatment determined by Western blotting. (C) Time course of p16 expression after Dox treatment (0.3 μg/mL) with IF staining (five randomly selected fields each with 1244, 487, 653, 601, and 561 cells for −Dox, 8, 16, 24, and 32 h +Dox, respectively). Three biological replicates were performed. Linear regression with 95% confidence interval is shown for p16 intensity. (D) Up‐regulation of p16 does not lead to change in p21, p53, phospho‐p53 (Ser15). Treatment with DNA damaging agent Doxorubicin is used as a control.

For temporal control of p16 expression in iPSCs and iPSC‐derived neurons, we used an inducible construct (pAAVS1‐p16) to express p16 under the tetracycline regulated TRE3G promoter. In the same construct, the reverse tetracycline‐controlled transactivator (rtTA) is constitutively expressed under the CAG promoter. We transfected the pAAVS1‐p16 construct into iPSCs for integration into the AAVS1 locus using a TALEN‐mediated gene targeting approach (Figure 1A) (Gonzalez et al. 2014; Mandegar et al. 2016). G418‐resistant iPSC clones were isolated and expanded from three genetic backgrounds: a familial AD (fAD) patient with the PSEN1‐A246E mutation, a sporadic AD (sAD) patient with the APOE4/E4 gene variant, and a healthy individual (Ctrl) without a history of dementia (Freibaum et al. 2015; Lavekar et al. 2023; Meyer et al. 2019). Colonies derived from each iPSC line were screened for successful on‐target integration into the AAVS1 locus by junction PCR (Figure S1A). The expression of p16 was induced by doxycycline (Dox) treatment for 2 days, and 0.3 μg/mL of Dox was found to achieve a high p16 expression level (Figure 1B). This Dox concentration (0.3 μg/mL) showed no obvious cytotoxicity as opposed to 1.0 μg/mL of Dox and was used for all experiments thereafter to minimize potential side effects of Dox. We treated pAAVS1‐p16‐iPSCs with Dox for various durations and found that 16 h of Dox treatment was sufficient to up‐regulate p16 expression in most cells (Figure 1C).

Next, we tested whether our inducible p16 system triggers the activation of the p53/p21 senescence pathway. In DNA damage response (DDR), p53 is phosphorylated and transactivates the expression of CDK inhibitor p21 (CDKN1A) to orchestrate cell cycle exit (d'Adda di Fagagna et al. 2003). While the DNA damaging agent doxorubicin increased p53 phosphorylation (p‐p53) at Serine 15 and p21 expression as expected, we observed that Dox treatment elevated only p16 expression but did not alter the levels of p‐p53 or p21, indicating that up‐regulation of p16 expression does not trigger the p53/p21 signaling (Figure 1D).

All established pAAVS1‐p16‐iPSC lines expressed pluripotency markers Sox2, Oct4, and Nanog (Figure S1A). One of the three iPSC lines (fAD with PSEN1 mutation) had a normal karyotype in 20 of 20 cells examined, while the other two lines (sAD with APOE4/E4 and Ctrl with no dementia) had abnormal karyotypes with an interstitial duplication in chromosome 20q in 6 and 9 of 20 cells examined, respectively (Figure S1B). This is a known recurrent duplication acquired at this location in human iPSC cultures (Laurent et al. 2011) and this abnormality does not affect p16 induction targeted at the AAVS1 locus on chromosome 19. The identity and purity of the isolated pAAVS1‐p16 lines were confirmed by STR analysis (Table S4).

### 3.2 Up‐Regulation of p16 Suppresses Cell Proliferation in iPSCs

**FIGURE 2:**
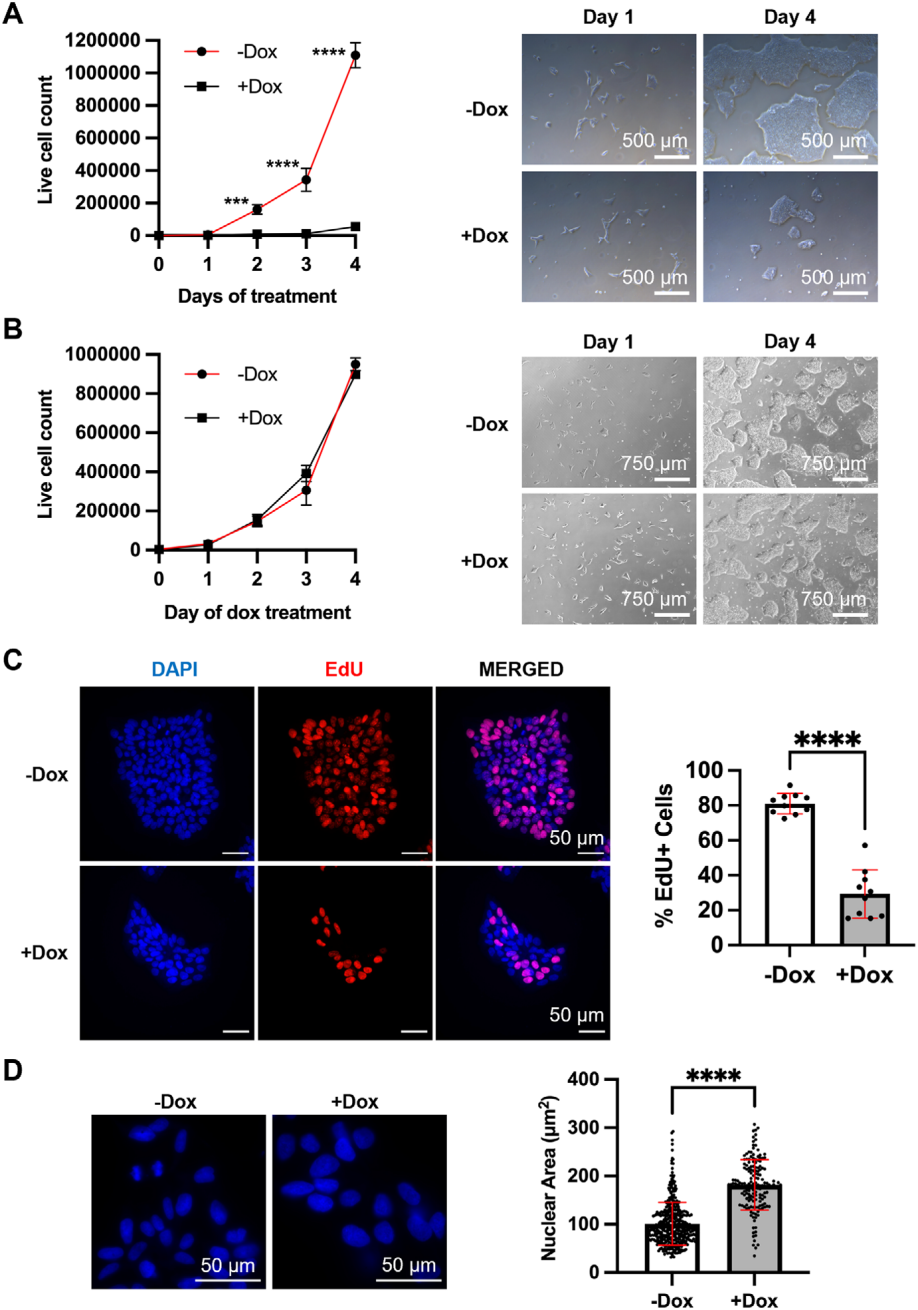
Up‐regulation of p16 suppresses cell proliferation in human iPSCs. Growth curves and brightfield images of iPSCs with 4‐day Dox treatment in (A) fAD line with AAVS1‐p16 showing suppression of cell proliferation and (B) fAD parental line without AAVS1‐p16 transgene showing no change in cell proliferation. (C) Reduced EdU incorporation after Dox treatment with IF staining (10 randomly selected fields each with 644 or 189 cells for −Dox or +Dox). (D) Increased nuclear size after Dox treatment (10 randomly selected fields each with 404 or 165 cells for −Dox or +Dox). Three biological and three technical replicates were performed. ***P < 0.001, ****P < 0.0001.

As a CDK inhibitor, p16 is known to limit cell proliferation (Serrano, Hannon, and Beach 1993). Consistently, we observed that Dox‐treated pAAVS1‐p16‐iPSCs grew significantly slower than untreated cells and formed smaller colonies after 4 days of Dox treatment (Figure 2A). Further, EdU incorporation was significantly decreased after 4–6 days of Dox treatment (Figures 2C and S2A,B). Dox treatment in parental iPSCs without the transfected pAAVS1‐p16 construct did not result in a decrease in cell proliferation (Figure 2B) or EdU incorporation (Figure S2C), indicating that changes observed in pAAVS1‐p16‐iPSCs are due to p16 up‐regulation rather than Dox per se.

**FIGURE 3:**
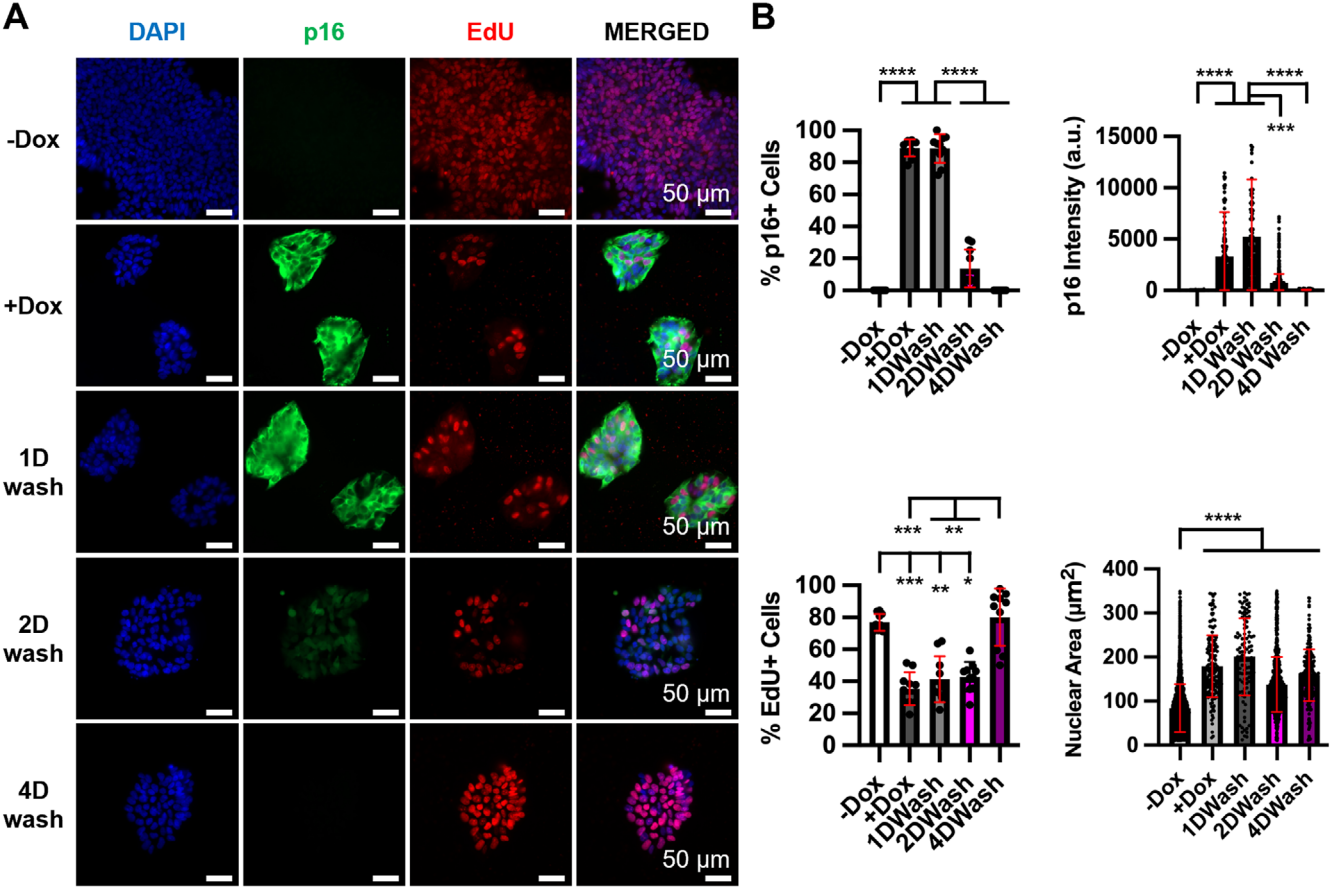
Removal of doxycycline leads to loss of p16 expression and restoration of cell proliferation but does not reduce nuclear size in iPSCs. (A) Representative IF staining and (B) quantitation (10 randomly selected fields each with 3150, 222, 153, 263, and 849 cells for −Dox, +Dox, 1D, 2D, or 4D wash). Two biological replicates were performed. *P < 0.05, **P < 0.01, ***P < 0.001, ****P < 0.0001.

We noticed that the nuclear size of Dox‐treated cells was increased compared to untreated cells (Figures 2D and S4B). As senescent cells often exhibit nuclear enlargement, we further investigated whether other senescence phenotypes are induced by p16 up‐regulation. Surprisingly, senescence‐associated β‐galactosidase (SA‐β‐gal) activity and loss of nuclear lamina protein Lamin B1 were not observed in Dox‐treated cells (Figure S4). This contrasts with what was reported previously that up‐regulation of p16 in human fibroblasts activates cellular senescence, including SA‐β‐gal and loss of Lamin B1 (Coppe et al. 2011; Freund et al. 2012; McConnell et al. 1998). To investigate whether continuous Dox treatment is required for p16 expression, we removed Dox from the culture after a 4‐day Dox treatment. Two days after Dox removal, p16 expression started to decrease significantly. Four days after Dox removal, p16 expression became undetectable (Figure 3). We found that EdU incorporation was increased when Dox was removed and p16 expression became undetectable (Figure 3), suggesting that proliferative arrest caused by p16 up‐regulation is reversible. Interestingly, nuclei remained enlarged after Dox removal and p16 expression was undetectable (Figure 3).

**FIGURE 4:**
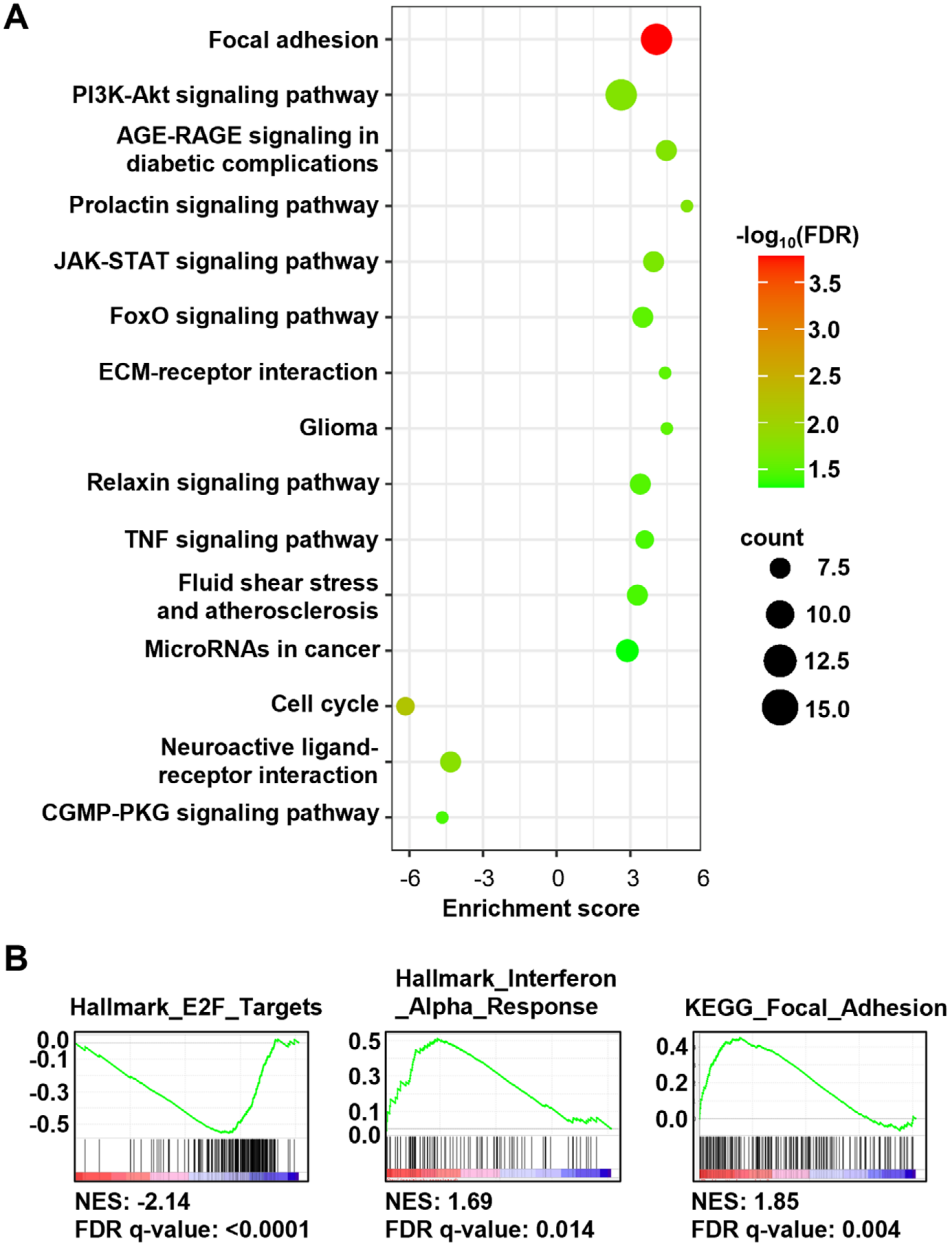
Up‐regulation of p16 results in alterations in genetic pathways including focal adhesion, cell cycle, and interferon α response in iPSCs. (A) Bubble plot showing enrichment of KEGG pathways of up‐regulated and down‐regulated DEGs after Dox‐treatment (FDR < 0.05). (B) Selected pathways enriched in GSEA using KEGG and Molecular Signatures Databases. Four biological replicates were performed.

To further delineate molecular changes following p16 induction, we performed RNA‐sequencing analysis of pAAVS1‐p16‐iPSCs treated with or without Dox for 2 or 4 days. Comparison of Dox‐treated iPSCs with untreated cells in fAD (with PSEN1 mutation) and Ctrl (without dementia) lines identified 544 differentially expressed genes (DEGs; FDR < 0.05, fold change > 1.5), with 382 genes up‐regulated and 162 genes down‐regulated in Dox‐treated cells (Table S5). Many down‐regulated genes are cell cycle genes such as PCNA, E2F2, MCM4, and MCM6 (Figure S5). Consistently, pathway enrichment analysis revealed that down‐regulated DEGs are enriched for pathways of cell cycle. Interestingly, up‐regulated DEGs were enriched for pathways of focal adhesion, PI3K‐Akt signaling, and extracellular matrix (ECM)‐receptor interaction (Figure 4A and Table S6). Further, we performed gene set enrichment analysis (GSEA) to explore potential biological functions. Using MSigDB hallmark and canonical KEGG pathway gene sets, we found that gene sets representing E2F targets, focal adhesion, and interferon alpha response were most enriched in Dox‐treated iPSCs (Figure 4B and Table S7) (Liberzon et al. 2015). As these genetic pathways are often enriched in senescent cells, we did GSEA with previously published gene signatures of senescence or senescence‐associated secretory phenotype (SASP) (Casella et al. 2019; De Cecco et al. 2019; Fridman and Tainsky 2008; Hernandez‐Segura et al. 2017; Jochems et al. 2021; Saul et al. 2022; Shah et al. 2013; Tasdemir et al. 2016) and found no significant enrichment of these gene signatures in pAAVS1‐p16‐iPSCs after Dox treatment (Table S7). Collectively, this inducible system of p16 expression in iPSCs robustly reduced cell proliferation and triggered some but not all phenotypes typically associated with cellular senescence.

### 3.3 Up‐Regulation of p16 in Neurons Differentiated From iPSCs Does Not Activate Senescence

**FIGURE 5:**
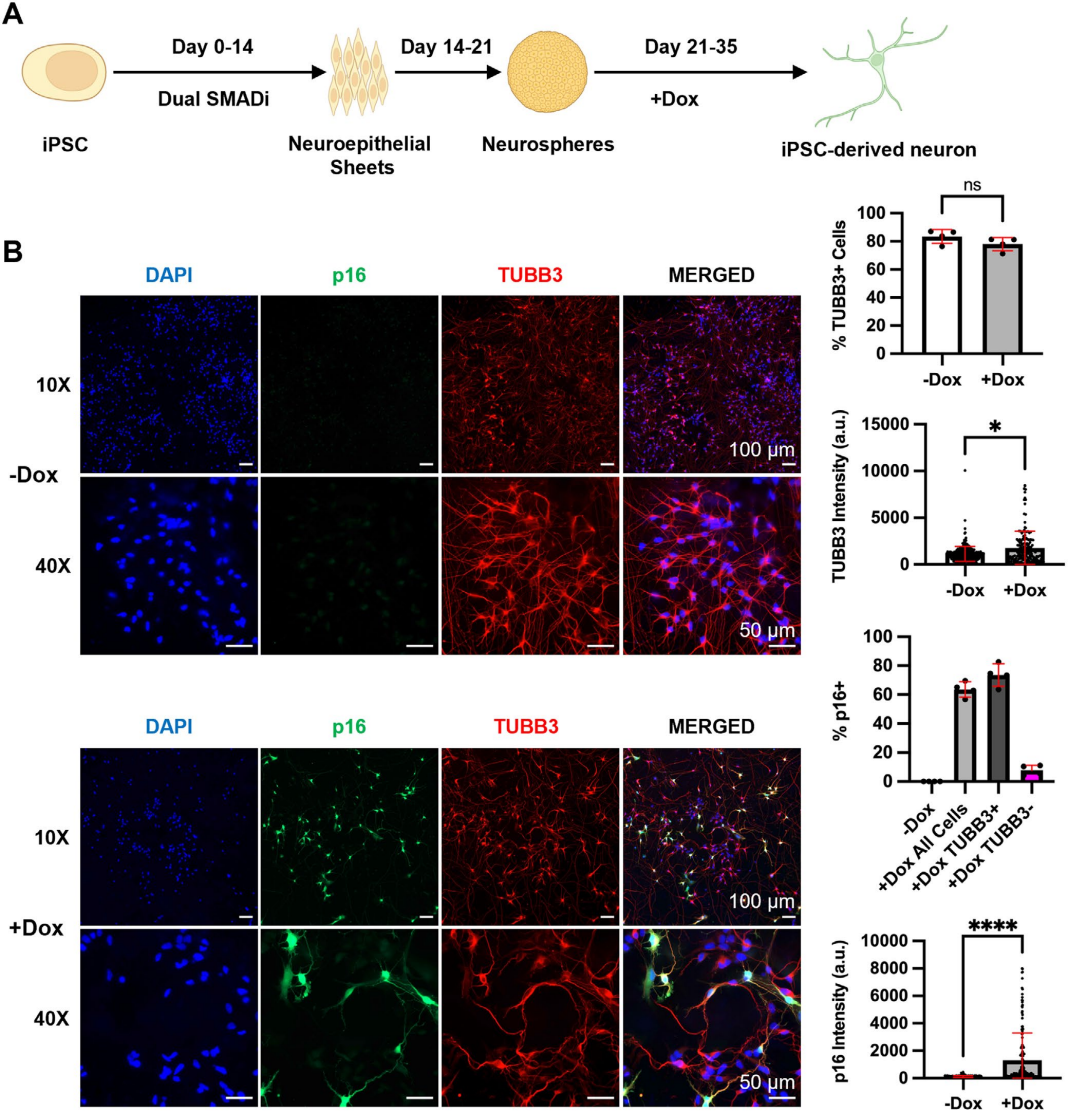
Doxycycline‐inducible expression of p16 in neurons differentiated from fAD iPSCs. (A) Outline of neuron differentiation protocol with Dox treatment during the last 2 weeks of differentiation. (B) Up‐regulation of p16 expression does not alter neuron differentiation with IF staining (four randomly selected fields each with 1521 or 773 cells for −Dox or +Dox). Three independent neuronal differentiation experiments each with 10 technical replicates were performed. *P < 0.05, ****P < 0.0001, ns: *P* > 0.05.

To determine whether our inducible pAAVS1‐p16 system is applicable in neural lineages, we used an established protocol for direct differentiation of iPSCs to cortical neurons (Cao et al. 2017; Chambers et al. 2009; Czerminski and Lawrence 2020) with Dox added to up‐regulate p16 expression in neurons (Figure 5A). Immunofluorescence staining showed that by day 14 of differentiation, no pluripotent (Oct4 and Nanog expressing) cells were detected, while nearly all cells stained positively for neural progenitor cells (NPCs) markers Pax6, Sox1, and Sox2, indicating they had differentiated into NPCs (Figure S6). Further differentiation (by day 35) led to efficient production of neurons: > 70% of the cells expressed neuronal markers TUBB3 or MAP2 (Figures 5B, S7 and S8). With 14‐day Dox treatment, p16 expression was up‐regulated and did not significantly alter the efficiency of neuron differentiation (Figures 5B, S7 and S8). About 47%–77% of the cells in the differentiated neuronal cultures expressed p16 with Dox treatment and most p16‐expressing cells were TUBB3+ neurons (Figures 5B and S8). The observation that not all neurons expressed p16 upon Dox treatment is consistent with prior reports of transgene silencing in differentiated cells, preferably neurons (Guillaume et al. 2006; Hong et al. 2007; Muotri et al. 2005; Qian et al. 2014). Overall, these results indicate that our inducible system is reliable in generating a “mosaic” culture to study the functional consequence of p16 up‐regulation in a subset of differentiated neurons.

Characteristics of cellular senescence including increased p16 and SA‐β‐gal have been reported in post‐mitotic neurons (Acklin et al. 2020; Herdy et al. 2022; Moreno‐Blas et al. 2019; Musi et al. 2018; Ohashi et al. 2018; Paramos‐de‐Carvalho et al. 2021; Raffaele et al. 2020; Riessland et al. 2019). However, whether p16 is sufficient to trigger senescence in non‐dividing neurons is unclear. Thus, we examined senescence‐related characteristics in Dox‐treated iPSC‐derived neurons. We did not observe a loss of Lamin B1, and instead Lamin B1 was slightly increased in Dox‐treated neurons (Figure S9). Further, Dox treatment did not significantly increase SA‐β‐gal activity or nuclear size in differentiated neurons (Figure S10), suggesting that up‐regulation of p16 expression does not activate cellular senescence in iPSC‐derived neurons.

### 3.4 Up‐Regulation of p16 Does Not Increase Aβ Secretion but Enhances Tau Phosphorylation in iPSC‐Derived Neurons

A hallmark of AD is the presence of amyloid plaques in the brain, which are extracellular aggregations of Aβ peptides from proteolytic processing of APP (Tanzi and Bertram 2005). APP is processed by the γ‐secretase complex to generate Aβ species of 37, 38, 40, 42, and 43 amino acids. Aβ40 is the most abundant in both healthy and AD brains, whereas Aβ42 has been shown to be a major component of plaques in AD and most likely the most deleterious (Tanzi and Bertram 2005). Previous studies revealed that p16 is expressed in cells within and surrounding Aβ plaques in the brains of AD mice and human AD patients (Arendt et al. 1996; Hu et al. 2021; Shin et al. 2024; Zhang et al. 2019). Thus, we investigated whether up‐regulation of p16 influences Aβ secretion in differentiated neurons. Using Meso Scale multiplex assays, we found that up‐regulation of p16 expression in iPSC‐derived neurons did not increase Aβ42 or Aβ40 secretion and Aβ42/40 ratio was largely unchanged in neurons differentiated from all 3 iPSC lines (Figure S11).

**FIGURE 6:**
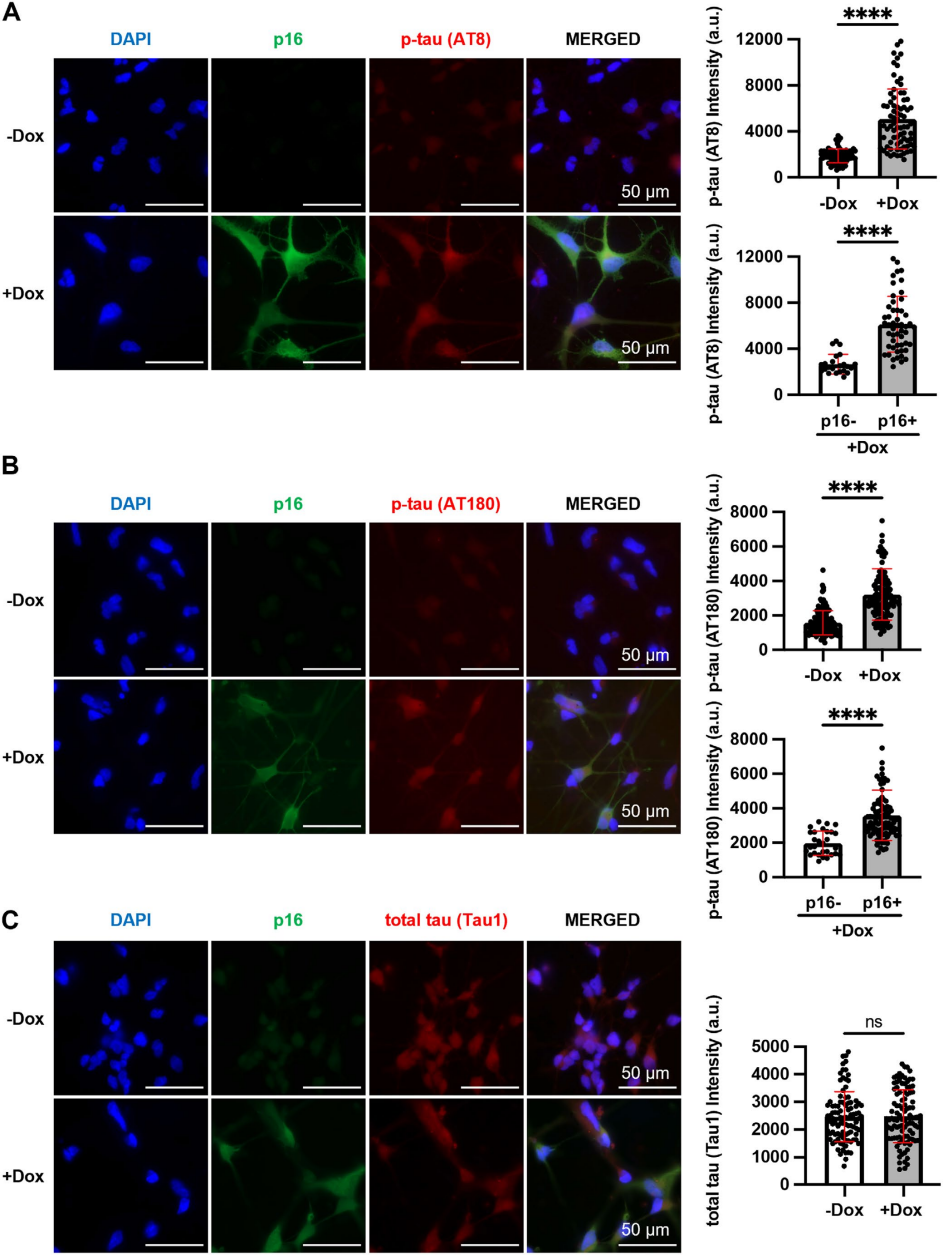
Up‐regulation of p16 enhances tau phosphorylation in neurons differentiated from fAD iPSCs. (A) Increased p‐tau (Ser202/Thr205 with AT8 antibody) upon p16 expression with IF staining (11 randomly selected fields each with 80 or 75 cells for −Dox or +Dox). (B) Increased p‐tau (Thr231 with AT180 antibody) upon p16 expression with IF staining (10 randomly selected fields each with 153 or 126 cells for −Dox or +Dox). (C) Unchanged total tau (with Tau1 antibody) level upon p16 expression with IF staining (10 randomly selected fields each with 98 or 101 cells for −Dox or +Dox). Three independent neuronal differentiation experiments each with 10 technical replicates were performed. ****P < 0.0001, ns: *P* > 0.05.

Abnormal tau phosphorylation underlies the formation of pathological NFTs, which correlate with AD severity of dementia (Arriagada et al. 1992). Previous studies have detected increased p16 expression in tau‐bearing neurons of AD patients (Arendt et al. 1996; McShea et al. 1997). Further, pathological tau tangles have been shown to induce p16 expression (Bussian et al. 2018; Musi et al. 2018). Importantly, the clearance of p16‐expressing cells in the brain ameliorates tau phosphorylation and improves cognitive function in an AD mouse model (Bussian et al. 2018). Nevertheless, studies have yet to determine whether p16 expression directly exacerbates tau phosphorylation in differentiated neurons. Thus, we examined phosphorylation status of tau protein at Ser202/Thr205 and Thr231 by IF staining in iPSC‐derived neurons upon p16 induction. Dox‐treated cells consistently showed significantly increased levels of phospho‐tau at Ser202/Thr205 and Thr231 compared to untreated cells (Figures 6A,B, S12 and S13). Further, the simultaneous presence of p16‐expressing and p16 negative cells in the same culture after Dox treatment allows us to compare these two populations of neurons side by side at the single cell level under the same culture and staining conditions. The p16‐expressing cells consistently showed significantly increased phospho‐tau levels at Ser202/Thr205 and Thr231 compared to p16 negative cells in the same Dox‐treated culture for all 3 lines (Figures 6A,B, S12 and S13), suggesting that p16 enhances tau phosphorylation in a cell‐autonomous manner. In contrast to observed change in phospho‐tau, total tau protein as detected by the Tau1 antibody was not altered by up‐regulation of p16 expression in differentiated neurons from all three lines (Figures 6C, S14). Meso Scale multiplex analysis also showed that Dox treatment does not significantly affect total tau protein secreted from differentiated neurons (Figure S14C). Collectively, these results suggest that increased phospho‐tau is not likely due to increased total tau or Aβ production but increased phosphorylation in differentiated neurons upon p16 induction.

## 4 Discussion

In this study, we generated an inducible system in human iPSCs to up‐regulate p16 expression temporally in a homogenous and synchronous manner. The versatility of human iPSCs allows us to precisely control the timing of p16 induction over the course of differentiation of iPSCs into desired cell types to study the functional consequences of p16 expression. Isogenic comparison of the same cell populations with or without p16 induction during differentiation of iPSCs reduces experimental heterogeneity in differentiated cultures. This approach offers tighter control over inter‐line variations compared to using differently manipulated isogenic cell lines, which are susceptible to epigenetic and genetic drift (Hall et al. 2008; Liang and Zhang 2013; Soldner and Jaenisch 2012). With this cellular system, we found that up‐regulation of p16 expression in differentiated neurons led to increased levels of phospho‐tau at Ser202/Thr205 and Thr231, while the level of total tau protein was not changed, suggesting that p16 expression results in enhanced phosphorylation of tau. To our knowledge, this is the first time that p16 has been found to play a role in the phosphorylation of tau. In contrast to tau, we did not observed changes in secreted Aβ peptides upon p16 induction. Further, the increase in tau phosphorylation was observed in all three genetic backgrounds, including two lines derived from human AD patients with genetic predisposition to AD and one line from a healthy individual without a history of dementia. This indicates that p16‐mediated changes in tau phosphorylation are a general consequence of p16 function independent of the disease background. Age is a major risk factor of AD, yet how aging contributes to AD is not known. As p16 expression is elevated during aging in many types of tissues and cells including brain and neurons (Herdy et al. 2022; Krishnamurthy et al. 2004), our study suggests that age‐associated up‐regulation of p16 could play an important role in AD by enhancing tau phosphorylation, thus providing a potential mechanism of aging contributing to AD pathology. In the brains of human AD patients and AD mouse models, p16 is often detected in neurons around amyloid plaques or in tau tangle‐bearing neurons (Arendt et al. 1996; Herdy et al. 2022; McShea et al. 1997; Musi et al. 2018; Wei et al. 2016). Aβ peptides or tau tangles have been found to induce p16 expression in neurons (Musi et al. 2018; Wei et al. 2016). Our finding of p16 increasing tau phosphorylation suggests a positive feed‐back loop, in which p16 increases tau phosphorylation and pathological tau tangles elevate p16 expression to further exacerbate pathogenesis. Moreover, we showed that tau phosphorylation was increased in neurons expressing p16 but not in cells without p16 expression in the same culture, suggesting a cell‐autonomous manner. Interestingly, several pathways including focal adhesion, interferon response, PI3K‐AKT, and JAK–STAT signaling, are enriched among the genes that were up‐regulated in p16‐expressing cells. It will be of great interest to further understand the underlying mechanisms of p16 leading to tau phosphorylation and whether any of the signaling pathways is involved.

Senescence is recently implicated to play an important role in AD pathogenesis (Arendt et al. 1996; Bhat et al. 2012; Bussian et al. 2018; Dehkordi et al. 2021; Gaikwad et al. 2021; Herdy et al. 2022; Hu et al. 2021; McShea et al. 1997; Musi et al. 2018; Streit et al. 2009; Zhang et al. 2019). It is possible that increased p16 expression as a part of the senescence program contributes to AD. It is interesting to note that neurons in AD brains are found to be prone to senescence (Herdy et al. 2022), and senescent neurons constitute the most senescent cells in AD brains (Dehkordi et al. 2021). The expression of p16 is frequently elevated in senescent cells, including senescent neurons (Baker et al. 2011; Herdy et al. 2022; Kang et al. 2015; Rayess, Wang, and Srivatsan 2012). Whether there is a functional consequence of p16 expression in the non‐dividing neurons during senescence is not clear. We found that p16 only activated some but not all senescence phenotypes, in contrast to what was observed in human fibroblasts (Coppe et al. 2011; Freund et al. 2012; McConnell et al. 1998). This discrepancy may reflect the heterogeneity of senescence observed among different cell types (Sharpless and Sherr 2015; van Deursen 2014). Even in human fibroblasts, increased expression of p16 results in many characteristic features of senescence, but an important senescence feature SASP is not activated by p16 (Coppe et al. 2011). We found no evidence of SASP upon p16 up‐regulation in iPSCs (Table S7). Previous studies have reported resistance in human embryonic stem cells and their derivatives to p16‐mediated cell cycle arrest and senescence (Dabelsteen et al. 2009; Ruiz et al. 2011). Our data suggests that p16 up‐regulation alone may not overcome the robust pluripotency network to trigger all the senescence phenotypes in iPSCs and their derivatives. It is also possible that with longer Dox treatment, other senescence phenotypes may be activated by p16. In iPSCs, doxycycline treatment led to p16 expression in ~90% of cells (Figure 1C). However, cells without p16 induction continued to grow and started to take over the culture after 5–6 days, limiting the duration of doxycycline treatment. Whether our results suggest a distinct function of p16 in regulation of tau phosphorylation independent of its role in senescence needs further investigation. In addition to neurons, the expression of p16 has been found to increase in astrocytes, microglia, and oligodendrocyte precursor cells in the brain tissues of human AD patients and mouse AD models (Arendt et al. 1996; Bhat et al. 2012; Bussian et al. 2018; Herdy et al. 2022; Musi et al. 2018; Wei et al. 2016; Zhang et al. 2019). Further, selective clearing of p16‐expressing cells with a genetic approach or removing senescent cells with senolytic compounds in AD mouse models have been shown to ameliorates various aspects of AD pathologies including tau phosphorylation, NFTs formation, Aβ load, and cognitive decline (Bussian et al. 2018; Hu et al. 2021; Musi et al. 2018; Zhang et al. 2019). It will be of great interest to use our system to differentiate iPSCs to astrocytes, microglia or other relevant cell types to directly investigate the impact of p16 on the AD‐related pathologies.

Reprogramming of somatic cells into iPSCs resets the epigenetic landscapes that mark the aging clock (Lapasset et al. 2011; Mahmoudi and Brunet 2012; Marion et al. 2009; Rando and Chang 2012), and consequently cells differentiated from iPSCs resemble fetal cells rather than adult or aged cells (Guttikonda et al. 2021; Israel et al. 2012; Jiang, Turkalj, and Xu 2020; Mariani et al. 2012; Miller et al. 2013; Nicholas et al. 2013; Patterson et al. 2012). Many efforts have been made to introduce or retain proper cellular aging in cell‐based modeling of age‐associated diseases. Strategies such as prolonged culture, expression of premature aging‐causing progeria protein, chemically induced cellular senescence or telomere shortening have been used in attempt to accelerate aging of iPSC‐derived cells (Fathi et al. 2022; Miller et al. 2013; Murray et al. 2015; Vera, Bosco, and Studer 2016; Zhang et al. 2015). It remains to be determined whether these methods trigger phenotypes that accurately mimic physiological and pathological aging. Direct reprogramming of fibroblasts to neurons (iNeurons) bypasses the iPSC stage and retains age‐related cellular and transcriptomic signatures. However, iNeurons are limited by their relatively poor reprogramming efficiency, low yield of reprogrammed cells and lack of versatility of iPSC‐based differentiation into various cell types (Herdy et al. 2022; Mertens et al. 2015; Mertens et al. 2018; Victor et al. 2018). As aging is a complex biological process, numerous contributing causes present a significant challenge to model age‐related changes for age‐associated diseases (Lopez‐Otin et al. 2013; van Deursen 2014). There is a critical need to combine multiple aging‐relevant alterations to robustly introduce proper cellular aging in iPSC‐based modeling of age‐associated diseases. As a consistent biomarker of aging, p16 is increased in many aging tissues including the brain (Idda et al. 2020; Jeck, Siebold, and Sharpless 2012; Krishnamurthy et al. 2004), and is causally associated with decreased neurogenesis during aging (Molofsky et al. 2006). Selective clearing of cells expressing p16 has been shown to extend lifespan and ameliorate age‐associated debilitations (Baker et al. 2016, 2011; Bussian et al. 2018; Childs et al. 2016; Jeon et al. 2017). In addition to its known role in senescence, we found in this study that increased p16 results in change in the pathways of interferon alpha response and ECM interactions, which are intricately linked to the aging hallmark of altered intercellular communication (Almontashiri et al. 2013; Brauer et al. 2023; Brenner et al. 2020; Darbro, Schneider, and Klingelhutz 2005; Lopez‐Otin et al. 2013; Yu et al. 2015). Our finding raises an intriguing possibility of using increased p16 expression as a way of introducing cellular aging in disease modeling, which warrants further investigation.

## Author Contributions

**Hong Zhang** designed and supervised the study, analyzed data, provided resources, and wrote the manuscript. **Kristopher Holloway** designed and performed most experiments, analyzed the data, and wrote the manuscript. **Kashfia Neherin** performed experiments in neuron differentiation and analyzed the data. **Feng Chen** and **Li Ding** supervised the RNA‐seq experiments and edited the manuscript. **Kazuhito Sato** and **Andrew Houston** performed RNA‐seq data. **Yingduo Song** analyzed RNA‐seq data. All authors discussed the results and contributed to the final manuscript.

## Conflicts of Interest

The authors declare no conflicts of interest.

## Supporting information


**Figure S1.** Characterization and validation of iPSC lines with AAVS1‐p16 integration.

**Figure S2.** Up‐regulation of p16 inhibits cell proliferation in human iPSCs.

**Figure S3.** Representative cell segmentation with Cell Profiler showing images used in (A) Figure 1C, (B) Figure 2C and (C) Figure S2.

**Figure S4.** Up‐regulation of p16 results in enlarged nuclei but does not lead to changes in SA‐β‐gal activity or Lamin B1 in iPSCs.

**Figure S5.** Up‐regulation of p16 in iPSCs leads to significant changes (FDR < 0.05, fold change > 1.5) in expression of genes involved in focal adhesion, PI3K‐Akt signaling (up‐regulated), or cell cycle (down‐regulated).

**Figure S6.** Efficient differentiation of iPSCs to NPCs. Representative IF staining of NPC markers Pax6, Sox1, Sox2, and pluripotency markers Oct3/4 and Nanog at day 14 of differentiation (10 randomly selected fields each with 529, 475, or 582 cells for Pax6, Sox1, or Sox2 staining).

**Figure S7.** Up‐regulation of p16 does not affect neuron differentiation from iPSCs.

**Figure S8.** Neuron differentiation from iPSCs is not altered by p16 expression.

**Figure S9.** Up‐regulation of p16 does not lead to loss of Lamin B1 in iPSC‐derived neurons.

**Figure S10.** Up‐regulation of p16 does not increase SA‐β‐gal activity or nuclear size in differentiated neurons.

**Figure S11.** Up‐regulation of p16 does not affect Aβ secretion in differentiated neurons.

**Figure S12.** Up‐regulation of p16 enhances tau phosphorylation at Ser202/Thr205 in iPSC‐derived neurons.

**Figure S13.** Up‐regulation of p16 enhances tau phosphorylation at Thr231 in iPSC‐derived neurons.

**Figure S14.** Up‐regulation of p16 does not change total tau level in iPSC‐derived neurons.

**Table S1.** Primer sequences for pAAVS1‐p16.
**Table S2.** Primer sequences for junction PCR.
**Table S3.** Short tandem repeat (STR) analysis of pAAVS‐p16 iPSCs.
**Table S4.** Antibodies used in immunofluorescence (IF) and Western blot.

**Table S5.** Differentially expressed genes in iPSCs after Dox treatment with FDR < 0.05 and fold change > 1.5.

**Table S6.** Significantly (FDR < 0.05) enriched KEGG and Hallmark MSigDB pathways in DEGs identified in iPSCs after Dox treatment.

**Table S7.** GSEA of MSigDB hallmark, KEGG, and senescence gene sets in iPSCs after Dox treatment with FDR < 0.05.

## Data Availability

RNA sequence data are deposited in Gene Expression Omnibus and the accession number is GSE252132. All reagents used in this study are commercially available. RNA sequencing data available at: https://www.ncbi.nlm.nih.gov/geo/query/acc.cgi?acc=GSE252132.
