# Supplementary figures and images for: Elevated p16Ink4a Expression Enhances Tau Phosphorylation in Neurons Differentiated From Human‐Induced Pluripotent Stem Cells
Source: Aging Cell. 2025 Jan 5;24(5):e14472. doi: 10.1111/acel.14472 (PMC12073902; doi:10.1111/acel.14472)

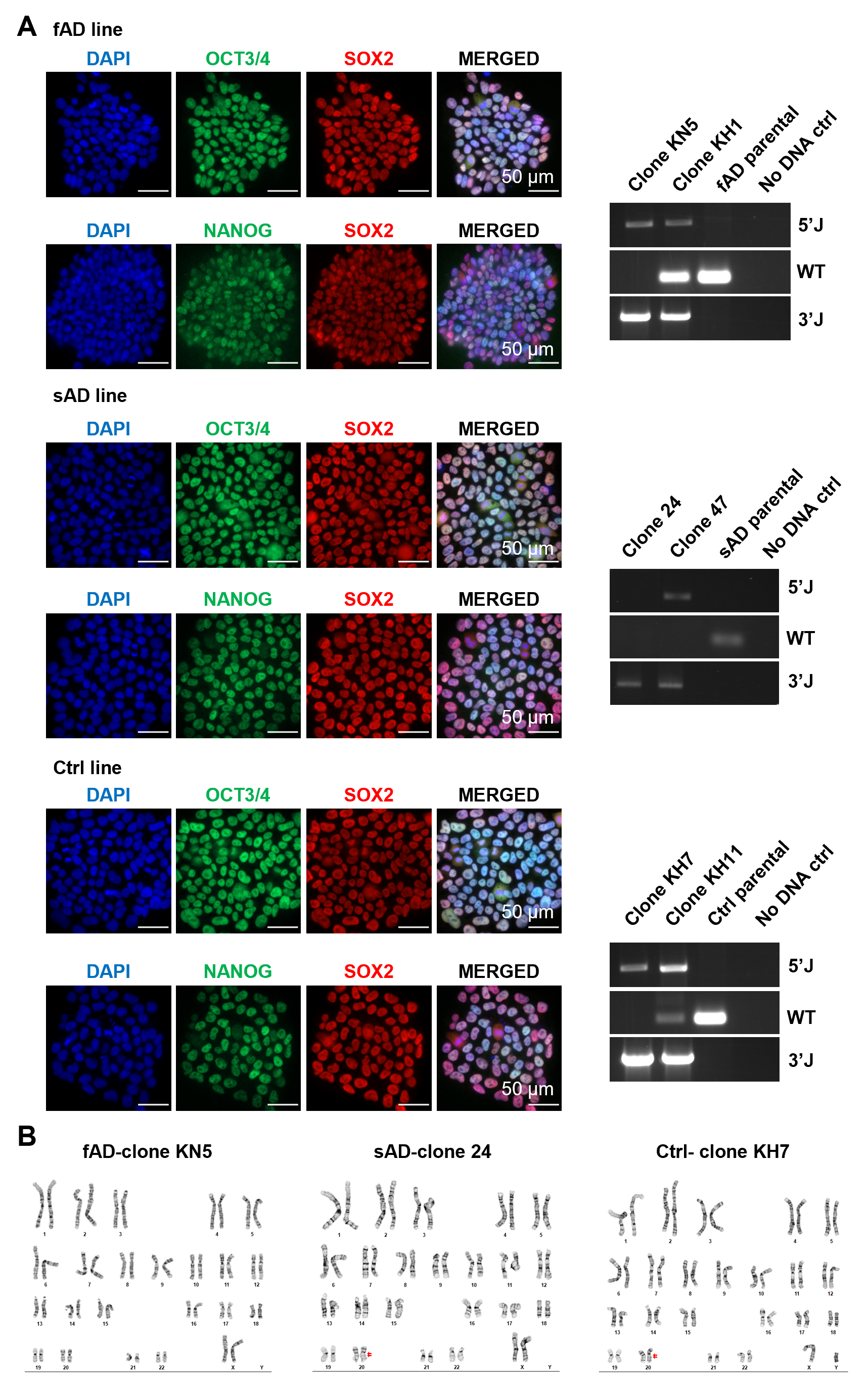

Supplement: Supplementary file 1 — Figure S1. Characterization and validation of iPSC lines with AAVS1‐p16 integration. [file ACEL-24-e14472-s016.png]

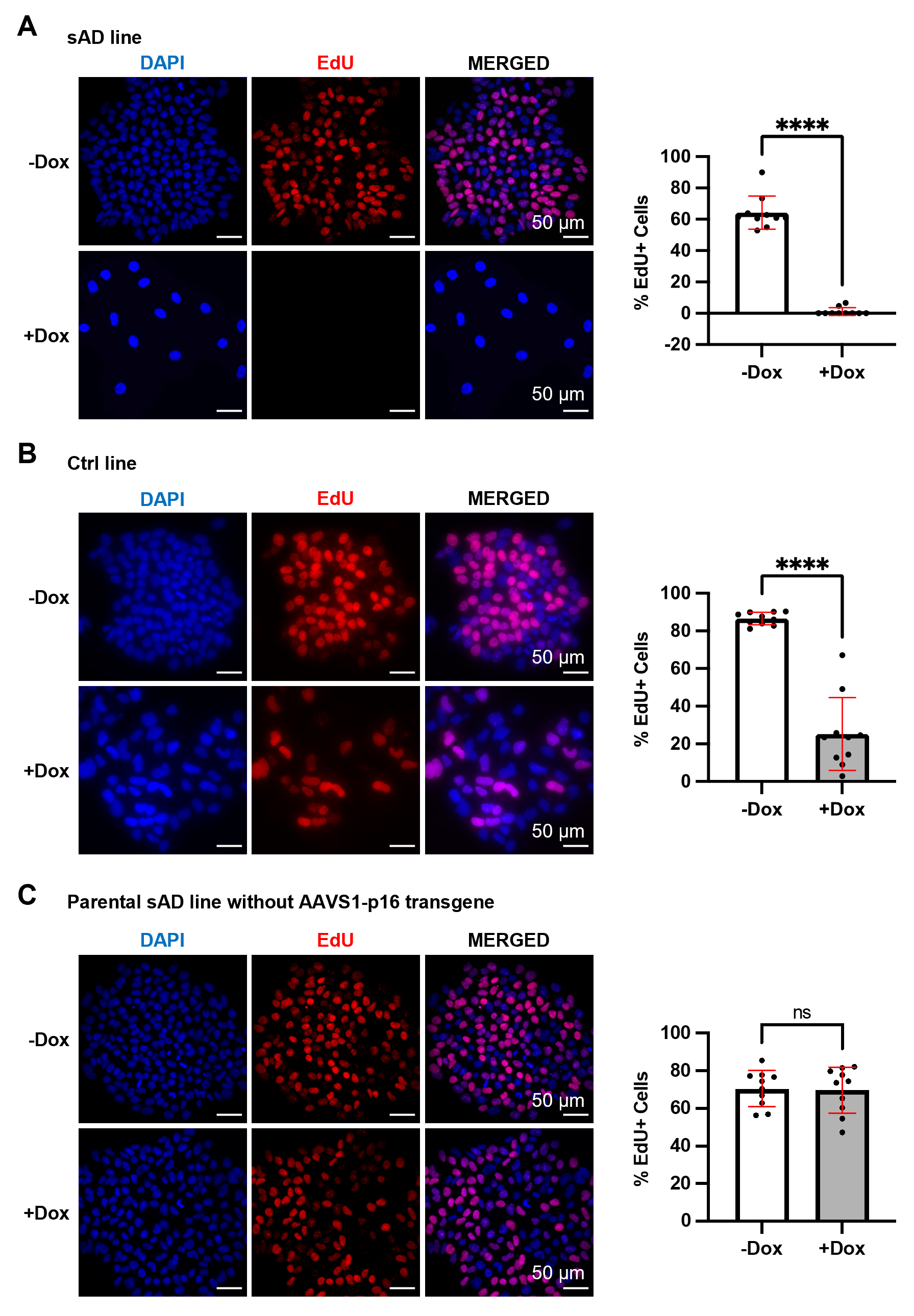

Supplement: Supplementary file 2 — Figure S2. Up‐regulation of p16 inhibits cell proliferation in human iPSCs. [file ACEL-24-e14472-s001.png]

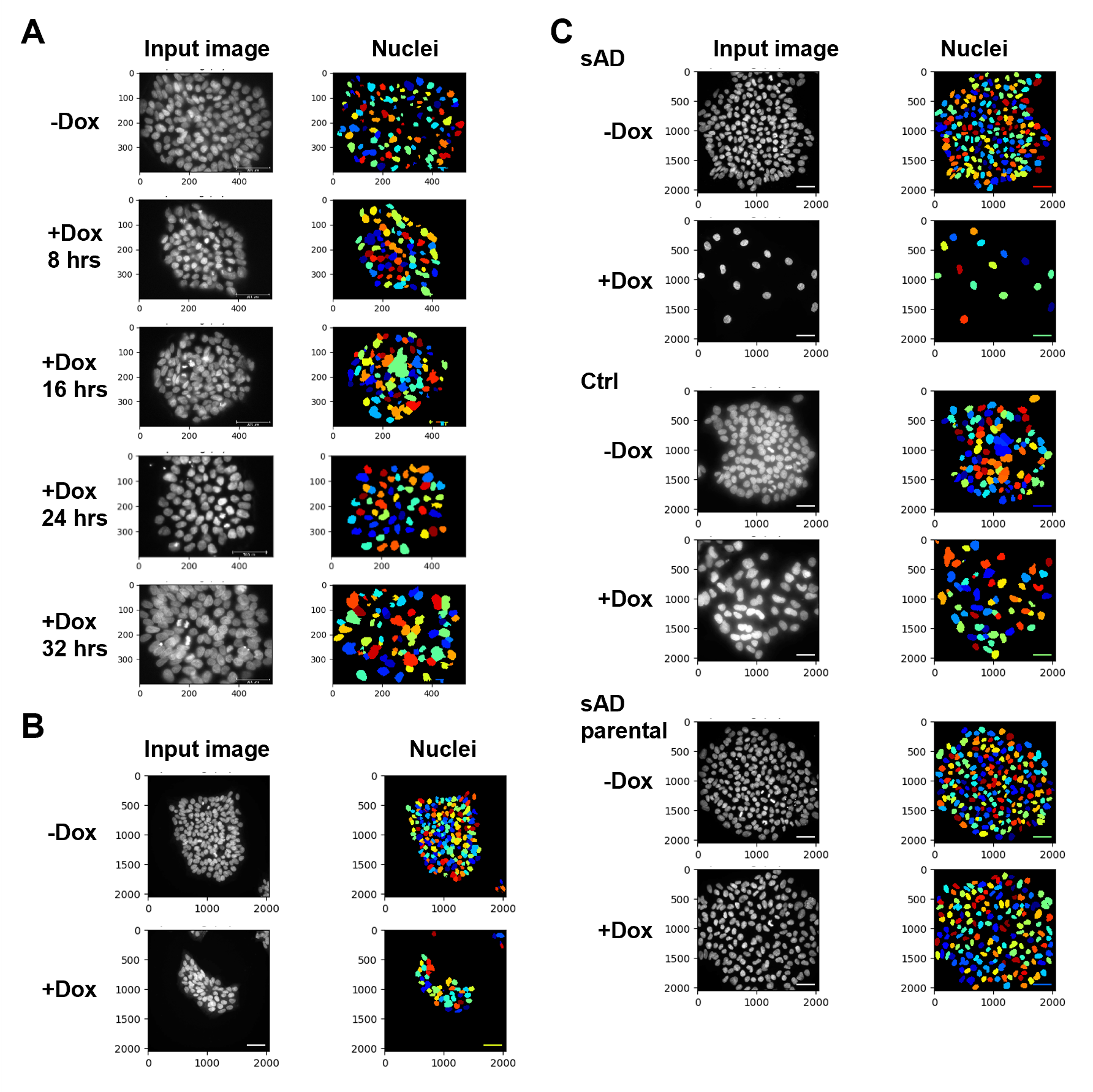

Supplement: Supplementary file 3 — Figure S3. Representative cell segmentation with Cell Profiler showing images used in (A) Figure 1C, (B) Figure 2C and (C) Figure S2. [file ACEL-24-e14472-s007.png]

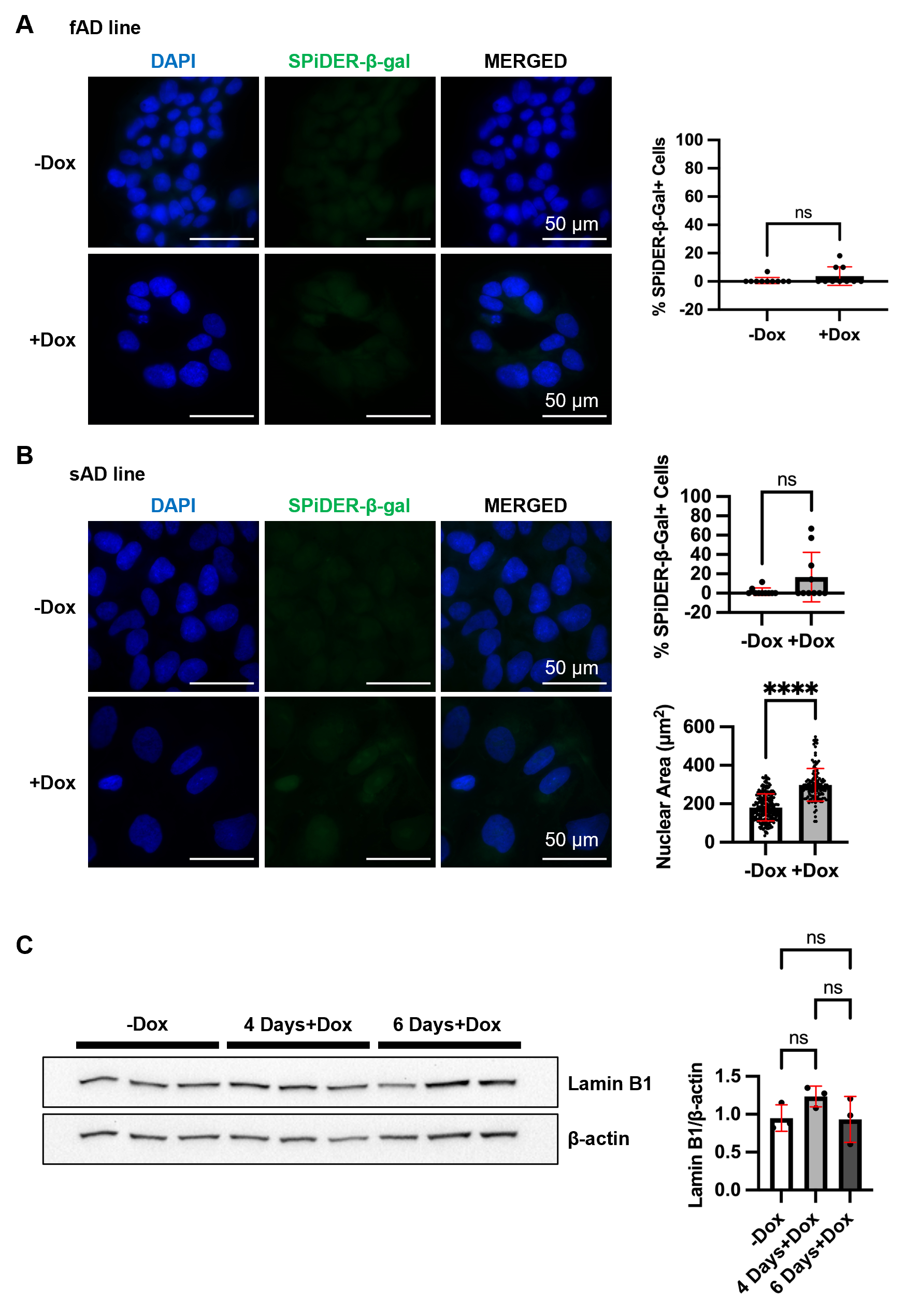

Supplement: Supplementary file 4 — Figure S4. Up‐regulation of p16 results in enlarged nuclei but does not lead to changes in SA‐β‐gal activity or Lamin B1 in iPSCs. [file ACEL-24-e14472-s018.png]

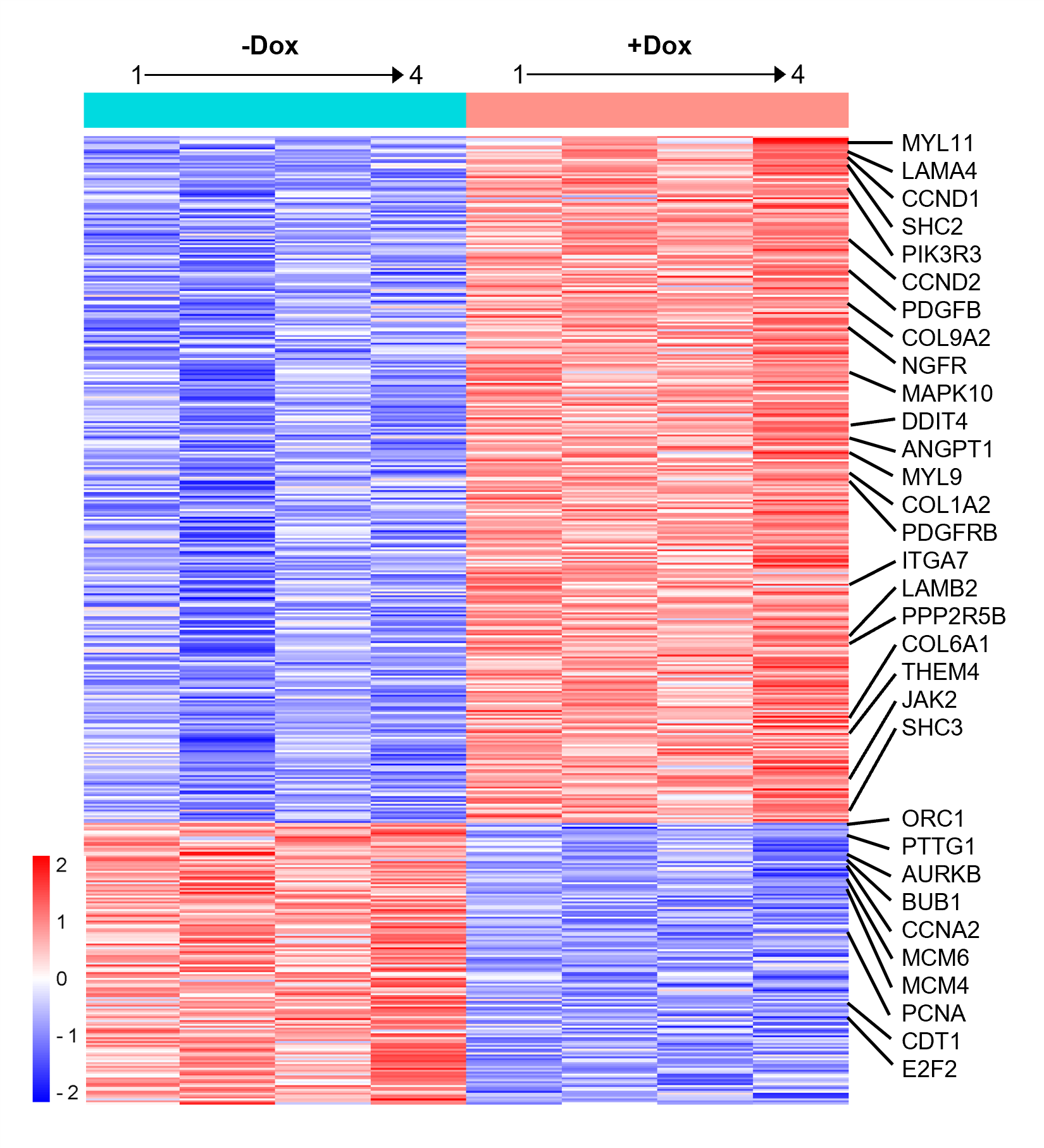

Supplement: Supplementary file 5 — Figure S5. Up‐regulation of p16 in iPSCs leads to significant changes (FDR < 0.05, fold change > 1.5) in expression of genes involved in focal adhesion, PI3K‐Akt signaling (up‐regulated), or cell cycle (down‐regulated). [file ACEL-24-e14472-s013.png]

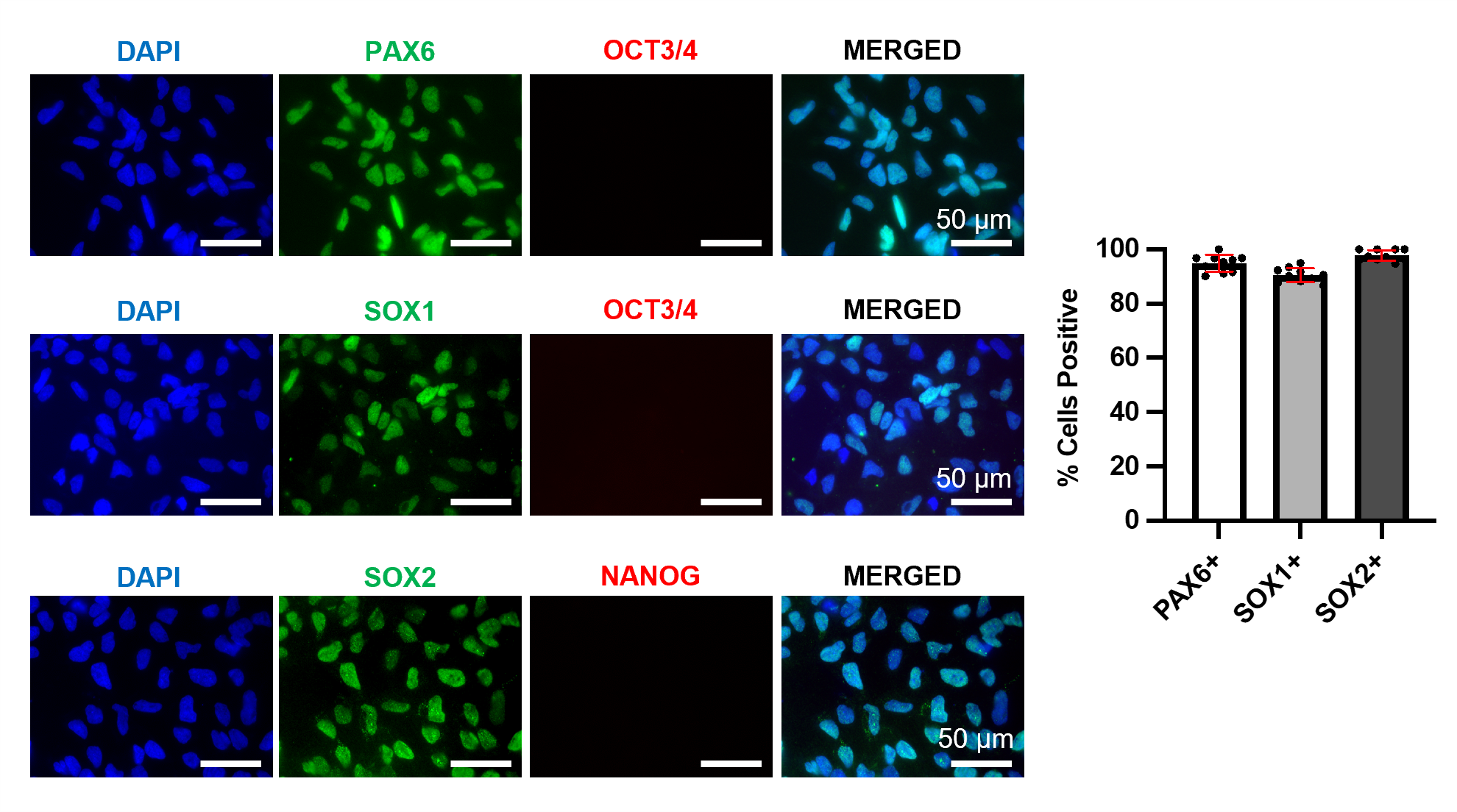

Supplement: Supplementary file 6 — Figure S6. Efficient differentiation of iPSCs to NPCs. Representative IF staining of NPC markers Pax6, Sox1, Sox2, and pluripotency markers Oct3/4 and Nanog at day 14 of differentiation (10 randomly selected fields each with 529, 475, or 582 cells for Pax6, Sox1, or Sox2 staining). [file ACEL-24-e14472-s011.png]

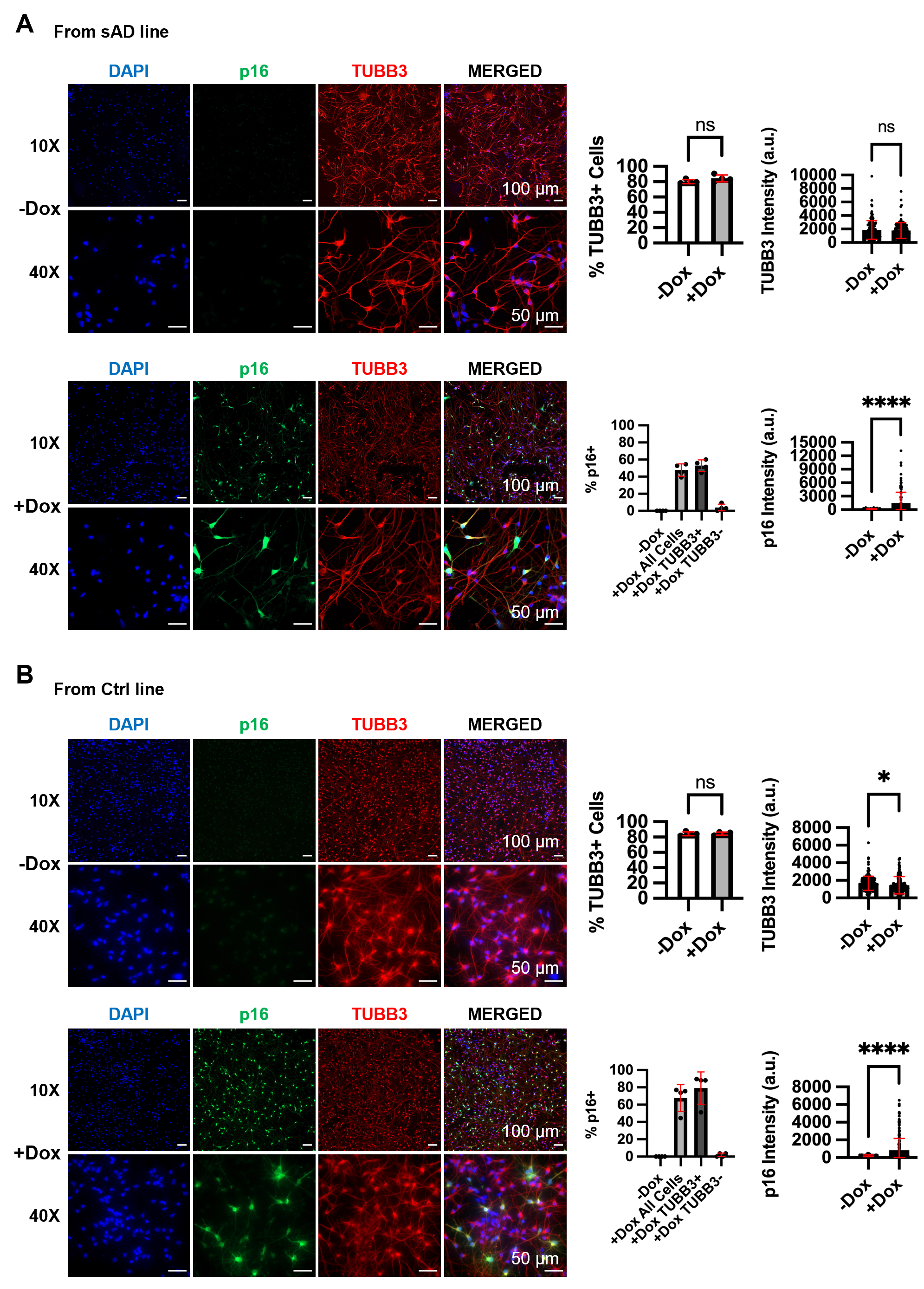

Supplement: Supplementary file 7 — Figure S7. Up‐regulation of p16 does not affect neuron differentiation from iPSCs. [file ACEL-24-e14472-s004.png]

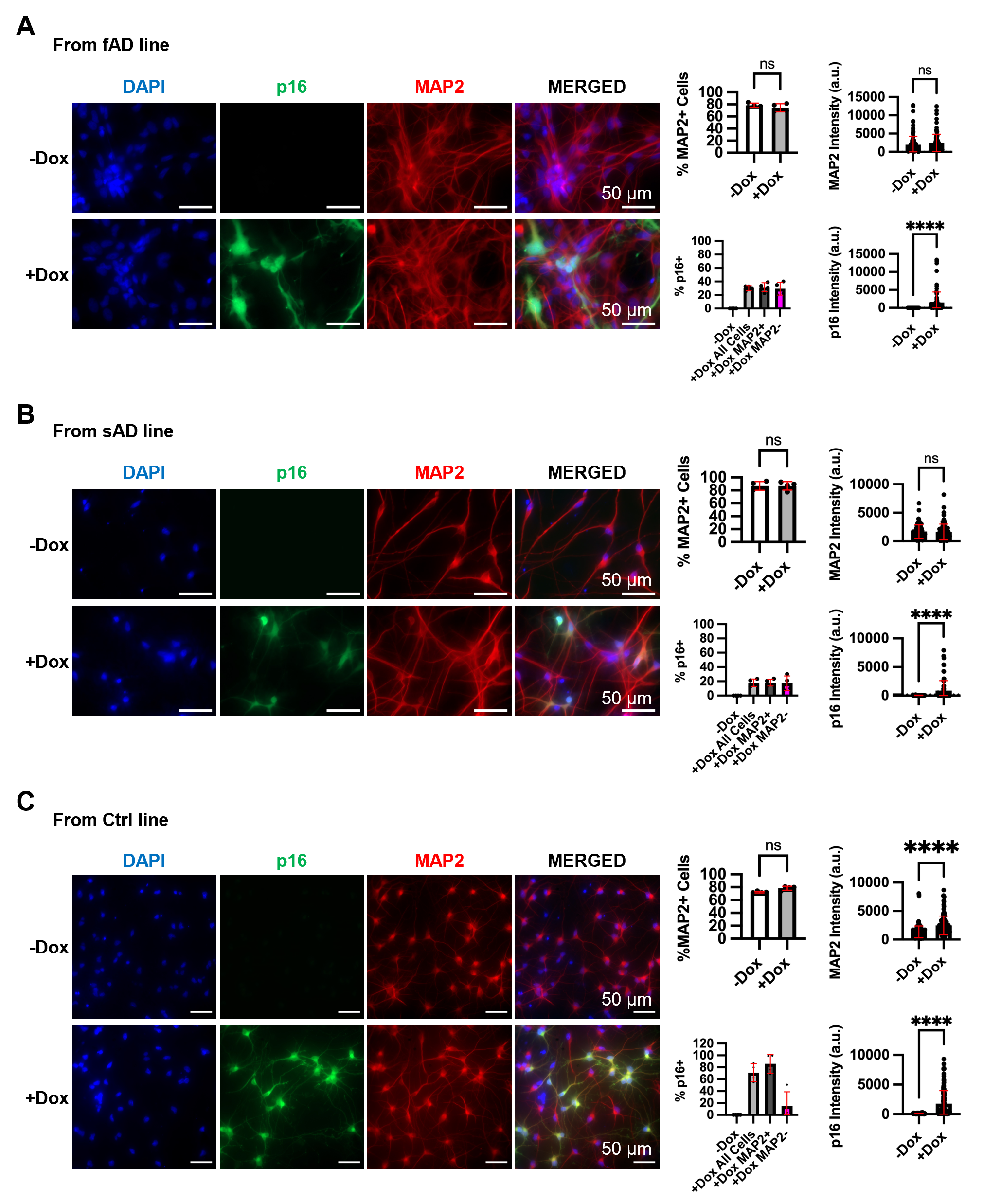

Supplement: Supplementary file 8 — Figure S8. Neuron differentiation from iPSCs is not altered by p16 expression. [file ACEL-24-e14472-s010.png]

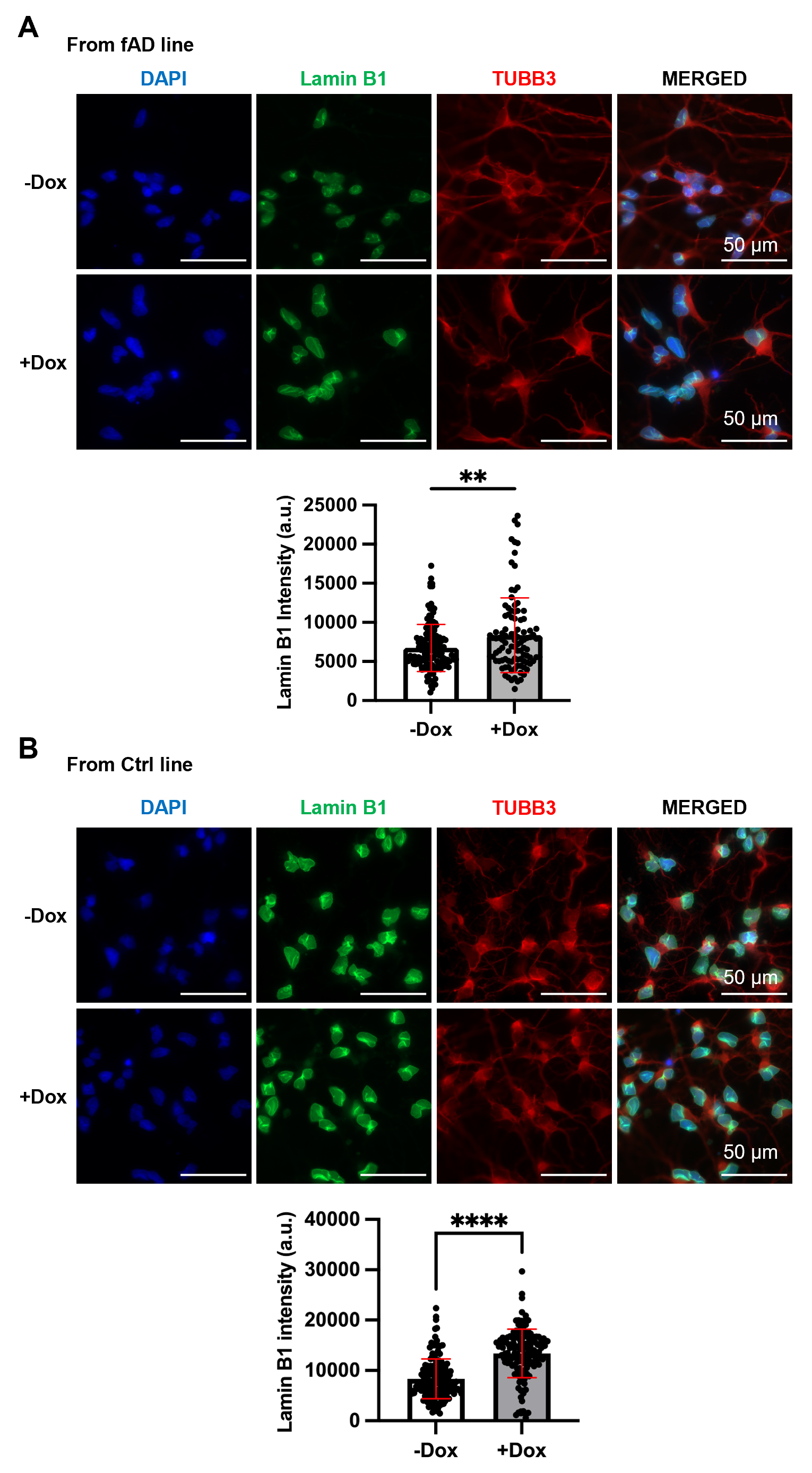

Supplement: Supplementary file 9 — Figure S9. Up‐regulation of p16 does not lead to loss of Lamin B1 in iPSC‐derived neurons. [file ACEL-24-e14472-s015.png]

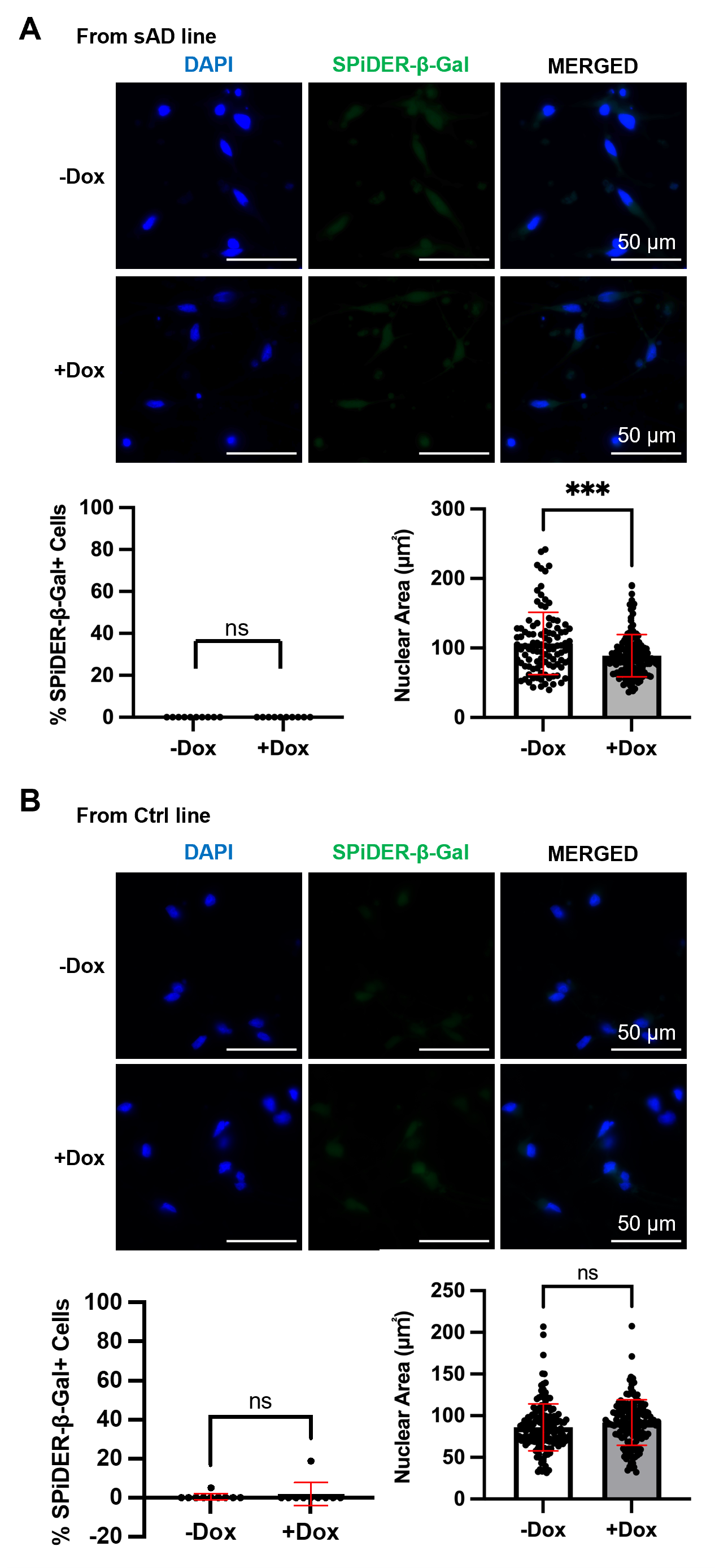

Supplement: Supplementary file 10 — Figure S10. Up‐regulation of p16 does not increase SA‐β‐gal activity or nuclear size in differentiated neurons. [file ACEL-24-e14472-s017.png]

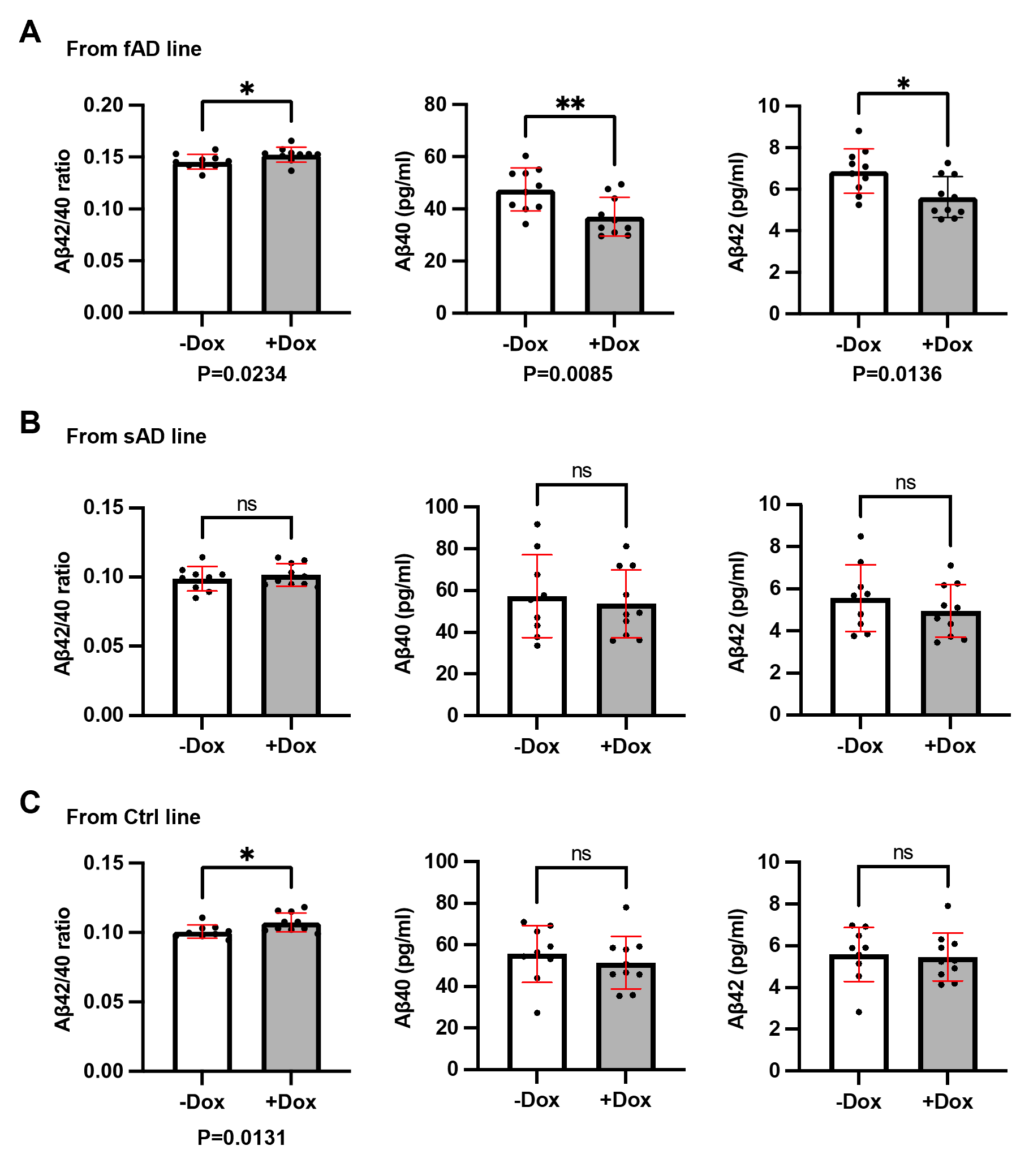

Supplement: Supplementary file 11 — Figure S11. Up‐regulation of p16 does not affect Aβ secretion in differentiated neurons. [file ACEL-24-e14472-s008.png]

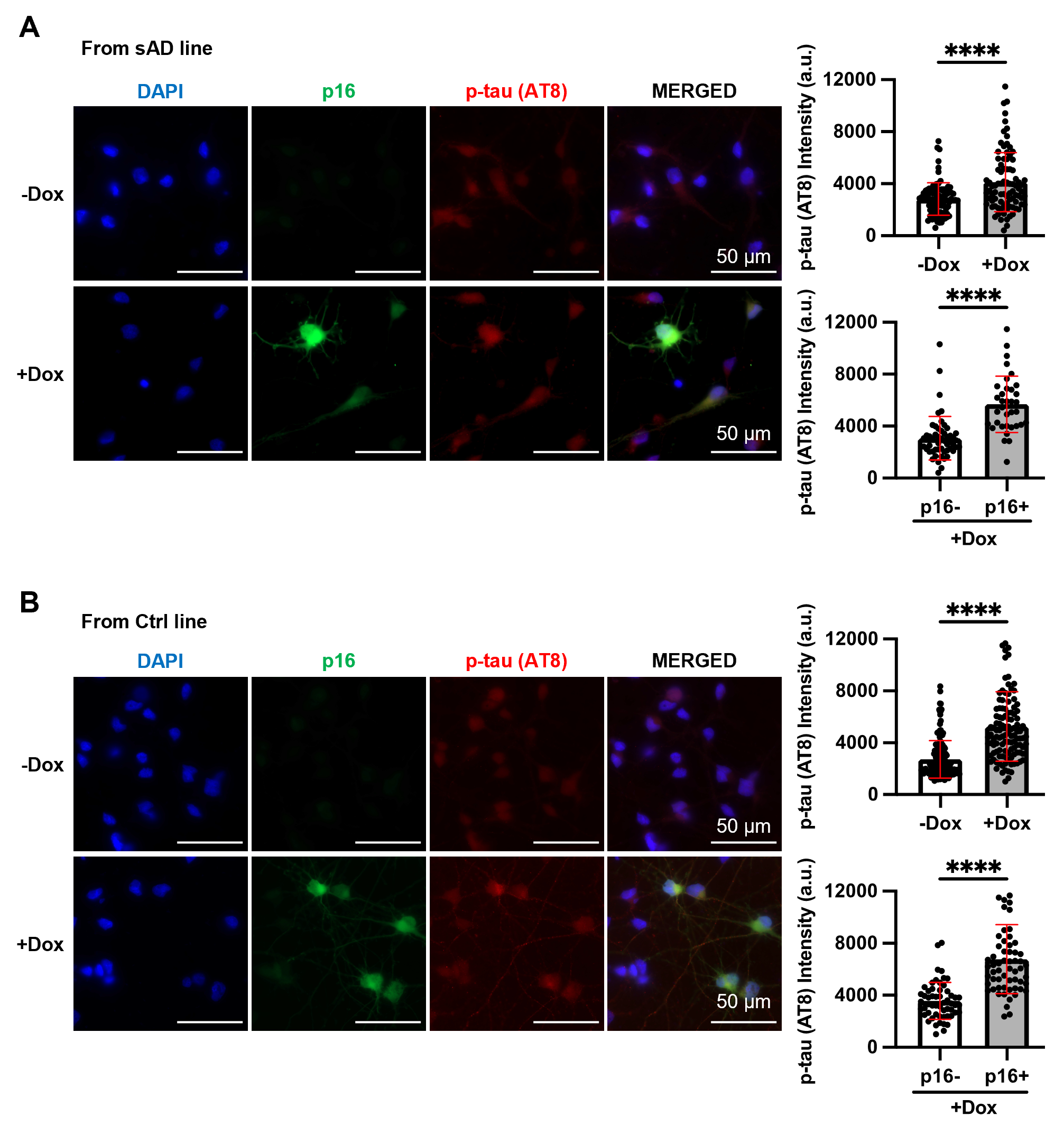

Supplement: Supplementary file 12 — Figure S12. Up‐regulation of p16 enhances tau phosphorylation at Ser202/Thr205 in iPSC‐derived neurons. [file ACEL-24-e14472-s014.png]

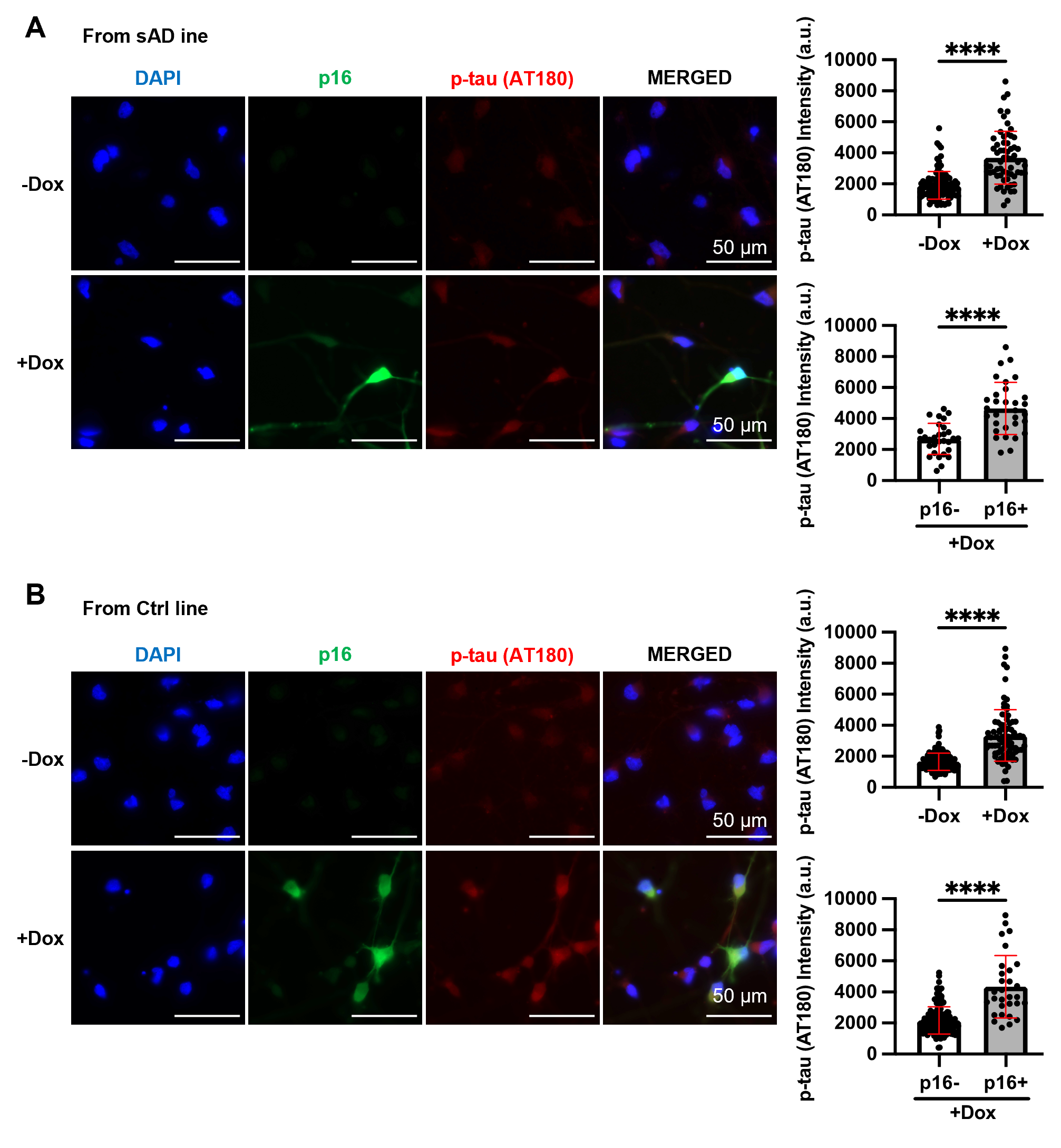

Supplement: Supplementary file 13 — Figure S13. Up‐regulation of p16 enhances tau phosphorylation at Thr231 in iPSC‐derived neurons. [file ACEL-24-e14472-s006.png]

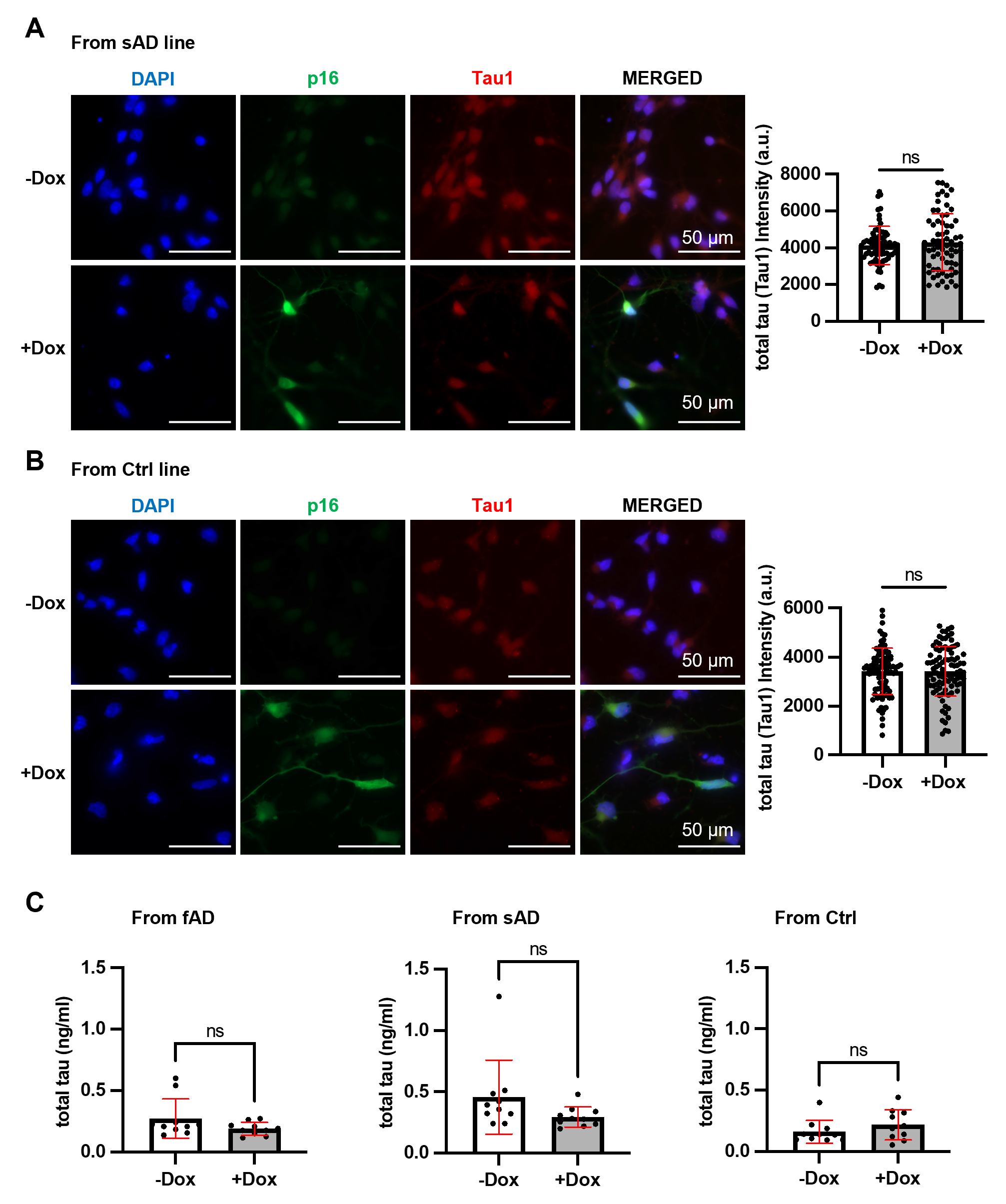

Supplement: Supplementary file 14 — Figure S14. Up‐regulation of p16 does not change total tau level in iPSC‐derived neurons. [file ACEL-24-e14472-s002.png]
