# Supplementary material for: Elevated p16Ink4a Expression Enhances Tau Phosphorylation in Neurons Differentiated From Human‐Induced Pluripotent Stem Cells
Source: Aging Cell. 2025 Jan 5;24(5):e14472. doi: 10.1111/acel.14472 (PMC12073902; doi:10.1111/acel.14472)
Supplement: Supplementary file 15 — Table S1. Primer sequences for pAAVS1‐p16. Table S2. Primer sequences for junction PCR. Table S3. Short tandem repeat (STR) analysis of pAAVS‐p16 iPSCs. Table S4. Antibodies used in immunofluorescence (IF) and Western blot. [file ACEL-24-e14472-s009.docx]

**Table S1.** Primer sequences for pAAVS1-p16.

| **Primer** | **Primer sequence (5’- 3’)** | **Notes** |
| --- | --- | --- |
| KHp16-F | GTTTAATTAAGCCACCATGGAACCCGCTGCTGGGTC | Amplifies p16 cDNA. Expected size is 469 bp. |
| KHp16-R | GTACCGGTTCAATCAGGAATATCTGATGGAC |  |

**Table S2.** Primer sequences for junction PCR.

| **Primer** | **Primer sequence (5’- 3’)** | **Notes** |
| --- | --- | --- |
| AAVS1 WT-F | CGGTTAATGTGGCTCTGGTT | Amplifies the WT AAVS1 locus. Expected size is 254 bp. |
| AAVS1 WT-R | AGGATCCTCTCTGGCTCCAT |  |
| AAVS1 5’-J-F | CTGCCGTCTCTCTCCTGAGT | Amplifies the 5’ integration junction. Expected size is 1258 bp. |
| Neo-R | CTCGTCCTGCAGTTCATTCA |  |
| TRE3G-F | GCGATCTGACGGTTCACTAAAC | Amplifies the 3’ integration junction. Expected size is 1376 bp. |
| AAVS1 3’-J-R | CCTGGGATACCCCGAAGAGT |  |

**Table S3.** Short tandem repeat (STR) analysis of pAAVS-p16 iPSCs.

**fAD (AG25367)**

| **Label on tube** | **Clone KN5**  **p40** | **AG25367 parental p28** |
| --- | --- | --- |
| **Label on Report** | 86200 | 78532 |
| **FGA** | 20, 22.2 | 20, 22.2 |
| **TPOX** | 8, 8 | 8, 8 |
| **D8S1179** | 13, 13 | 13, 13 |
| **vWA** | 18, 18 | 18, 18 |
| **Amelogenin** | X, X | X, X |
| **Penta_D** | 9, 13 | 9, 13 |
| **CSF1PO** | 12, 12 | 12, 12 |
| **D16S539** | 9, 12 | 9, 12 |
| **D7S820** | 8, 10 | 8, 10 |
| **D13S317** | 11, 11 | 11, 11 |
| **D5S818** | 11, 12 | 11, 12 |
| **Penta_E** | 11, 14 | 11, 14 |
| **D18S51** | 13, 14 | 13, 14 |
| **D21S11** | 30, 30 | 30, 30 |
| **TH01** | 6, 9.3 | 6, 9.3 |
| **D3S1358** | 14, 17 | 14, 17 |
| **Allelic Polymorphisms** | 24 | 24 |
| **Matches** | 78532 | 86200 |

Results: The genotypic profiles are comprised of 24 allelic polymorphisms across the 15 STR loci analyzed.

Interpretation: The DNA concentration required to achieve an acceptable STR genotype (signal/ noise) was equivalent to that required for the standard procedure (~1 ng/amplification reaction) from human genomic DNA. These results suggest that the cells submitted correspond to the cell lines as named and were not contaminated with any other human cells or a significant amount of mouse feeder layer cells.

Sensitivity: Sensitivity limits for detection of STR polymorphisms unique to either this or other human cell lines is ~2-5%.

**sAD (AG27609)**

| **Label on tube** | **Clone 24**  **p53** | **AG27609 parental p45** |
| --- | --- | --- |
| **Label on Report** | 99577 | 100132 |
| **FGA** | 22, 23 | 22, 23 |
| **TPOX** | 8, 8 | 8, 8 |
| **D8S1179** | 9, 12 | 9, 12 |
| **vWA** | 17, 17 | 17, 17 |
| **Amelogenin** | X, X | X, X |
| **Penta_D** | 11, 11 | 11, 11 |
| **CSF1PO** | 10, 10 | 10, 10 |
| **D16S539** | 11, 12 | 11, 12 |
| **D7S820** | 10, 10 | 10, 10 |
| **D13S317** | 11, 12 | 11, 12 |
| **D5S818** | 12, 12 | 12, 12 |
| **Penta_E** | 12, 12 | 12, 12 |
| **D18S51** | 12, 21 | 12, 21 |
| **D21S11** | 28, 33.2 | 28, 33.2 |
| **TH01** | 8, 9 | 8, 9 |
| **D3S1358** | 17, 18 | 17, 18 |
| **Allelic Polymorphisms** | 23 | 24 |
| **Matches** | 100132 | 99577 |

Results: The genotypic profiles comprise a range of 23 allelic polymorphisms across the 15 STR loci analyzed.

Interpretation: The concentration of DNA required to achieve an acceptable STR genotype (signal/ noise) was equivalent to that required for the standard procedure (~1 ng/amplification reaction) from human genomic DNA. These results suggest that the cells submitted correspond to the cell lines as named and were not contaminated with any other human cells or a significant amount of mouse feeder layer cells.

Sensitivity: Sensitivity limits for detection of STR polymorphisms unique to either this or other human cell lines is ~2-4%.

**Ctrl (35L11)**

| **Label on tube** | **Clone KH7**  **p37** | **35L11 parental**  **p29** |
| --- | --- | --- |
| **Label on Report** | 99576 | 84288 |
| **FGA** | 22, 22 | 22, 22 |
| **TPOX** | 8, 9 | 8, 9 |
| **D8S1179** | 10, 14 | 10, 14 |
| **vWA** | 14, 17 | 14, 17 |
| **Amelogenin** | X, Y | X, Y |
| **Penta_D** | 8, 11 | 8, 11 |
| **CSF1PO** | 10, 12 | 10, 12 |
| **D16S539** | 10, 11 | 10, 11 |
| **D7S820** | 10, 10 | 10, 10 |
| **D13S317** | 11, 13 | 11, 13 |
| **D5S818** | 11, 11 | 11, 11 |
| **Penta_E** | 11, 17 | 11, 17 |
| **D18S51** | 11, 15 | 11, 15 |
| **D21S11** | 29, 30 | 29, 30 |
| **TH01** | 8, 9 | 8, 9 |
| **D3S1358** | 15, 16 | 15, 16 |
| **Allelic Polymorphisms** | 27 | 27 |
| **Matches** | 84288 | 99576 |

Results: The genotypic profiles comprise a range of 27 allelic polymorphisms across the 15 STR loci analyzed.

Interpretation: The concentration of DNA required to achieve an acceptable STR genotype (signal/ noise) was equivalent to that required for the standard procedure (~1 ng/amplification reaction) from human genomic DNA. These results suggest that the cells submitted correspond to the cell lines as named and were not contaminated with any other human cells or a significant amount of mouse feeder layer cells.

Sensitivity: Sensitivity limits for detection of STR polymorphisms unique to either this or other human cell lines is ~2-4%.

**Table S4.** Antibodies used in immunofluorescence (IF) and Western blot.

| **Antibodies** | **Species** | **Catalog no.** | **Company** | **Dilution** |
| --- | --- | --- | --- | --- |
| **IF** |  |  |  |  |
| Nanog | Mouse | MABD24 | Sigma | 1:100 |
| Oct3/4 | Mouse | sc-5279 | Santa Cruz Biotechnology | 1:100 |
| Sox2 | Goat | AF2018 | R&D Systems | 1:100 |
| p16 | Rabbit | ab108349 | Abcam | 1:500 |
| TUBB3 | Mouse | 801201 | Biolegend | 1:500 |
| Phospho-Tau Ser202/Thr205 (AT8) | Mouse | MN1020 | Invitrogen | 1:100 |
| Phospho-Tau  Thr231 (AT180) | Mouse | MN1040 | Invitrogen | 1:100 |
| Lamin B1 | Rabbit | ab16048 | Abcam | 1:500 |
| Sox1 | Rabbit | CST 4194 | Cell Signaling Technology | 1:100 |
| Pax6 | Rabbit | 901301 | BioLegend | 1:500 |
| MAP2 | Chicken | PA1-10005 | Invitrogen | 1:500 |
| **Western** |  |  |  |  |
| p16 | Rabbit | ab108349 | Abcam | 1:1000 |
| p21 | Rabbit | CST 2947 | Cell Signaling Technology | 1:1000 |
| p53 | Mouse | CST 2524 | Cell Signaling Technology | 1:1000 |
| p-p53 (Ser15) | Rabbit | CST 9284 | Cell Signaling Technology | 1:1000 |
| Lamin B1 | Rabbit | ab16048 | Abcam | 1:2000 |
| β-Actin | Rabbit | CST 8457 | Cell Signaling Technology | 1:1000 |
| Vinculin | Mouse | V9131 | Sigma | 1:10000 |
| **Secondary Abs** |  |  |  |  |
| Anti-mouse  Alexa fluor 488 | Donkey | A21202 | Invitrogen | 1:500 |
| Anti-mouse  Alexa fluor 594 | Goat | A11005 | Invitrogen | 1:500 |
| Anti-rabbit  Alexa fluor 488 | Goat | A11008 | Invitrogen | 1:500 |
| Anti-rabbit  Alexa fluor 594 | Goat | A11012 | Invitrogen | 1:500 |
| Anti-goat  Alexa fluor 594 | Donkey | A11058 | Invitrogen | 1:500 |
| Anti-chicken  Alexa fluor 594 | Goat | A11042 | Invitrogen | 1:500 |
| Anti-mouse HRP | Sheep | NA931 | Cytiva | 1:5000 |
| Anti-rabbit HRP | Donkey | NA934 | Cytiva | 1:5000 |
